# MaxMIF: A New Method for Identifying Cancer Driver Genes through Effective Data Integration

**DOI:** 10.1002/advs.201800640

**Published:** 2018-07-23

**Authors:** Yingnan Hou, Bo Gao, Guojun Li, Zhengchang Su

**Affiliations:** ^1^ School of Mathematics Shandong University Jinan 250100 P. R. China; ^2^ State Key Laboratory of Microbial Technology Shandong University Jinan 250100 P. R. China; ^3^ Department of Bioinformatics and Genomics The University of North Carolina at Charlotte 9201, University City Blvd Charlotte NC 28223 USA

**Keywords:** cancer driver gene prediction, candidate gene prioritization, functional networks, maximal mutational impact function, somatic mutations

## Abstract

Identification of a few cancer driver mutation genes from a much larger number of passenger mutation genes in cancer samples remains a highly challenging task. Here, a novel method for distinguishing the driver genes from the passenger genes by effective integration of somatic mutation data and molecular interaction data using a maximal mutational impact function (MaxMIF) is presented. When evaluated on six somatic mutation datasets of Pan‐Cancer and 19 datasets of different cancer types from TCGA, MaxMIF almost always significantly outperforms all the existing state‐of‐the‐art methods in terms of predictive accuracy, sensitivity, and specificity. It recovers about 30% more known cancer genes in 500 top‐ranked candidate genes than the best among the other tools evaluated. MaxMIF is also highly robust to data perturbation. Intriguingly, MaxMIF is able to identify potential cancer driver genes, with strong experimental data support. Therefore, MaxMIF can be very useful for identifying or prioritizing cancer driver genes in the increasing number of available cancer genomic data.

## 1 Introduction

Cancer is one of the most complex diseases that threaten human health. Systemic cancer genomics projects such as the Cancer Genome Atlas (TCGA)^1^ and the International Cancer Genome Consortium (ICGC)^2^ have produced and analyzed a large number of genomics data in various cancers, providing an unprecedented opportunity to elucidate the etiology of cancer.^3^ It has been observed that the gene mutation rate in cancer cells was significantly higher than that in normal cells,^4^ suggesting that gene mutation is closely related to cancer. However, cancer exhibits extensive mutational heterogeneity, resulting in the so‐called long‐tail phenomenon that a small number of mutated genes are seen in vast majority of cancers while a large number of other mutated genes are found in only few cancers.^5^ According to the driver–passenger model, driver mutations render a selective advantage to cancer cells, thereby promoting cancer occurrence, while passenger mutations play little role in driving cancer.^6^

A great deal of efforts have been made to distinguish drivers from passengers. Some methods such as Mutation_Assessor,^7^ CHASM,^8^ transFIC,^9^ and FATHMM^10^ predict possible driver mutations by assessing functional impact of missense mutations. Other methods such as MutSig2.0,^11^ MutSigCV,^12^ InVEx,^13^ and MuSiC^14^ predict as possible driver genes those with extraordinary higher mutation rates than background mutation rates (BMR). A considerable number of genes have been identified as driver genes by these methods. Unfortunately, due to the long‐tail phenomenon, methods based on mutation frequency are underpowered for uncovering infrequently mutated driver genes. The observation that mutations in a cancer genome tend to converge on a few biological pathways,^15^ has prompted the development of pathway‐based or network‐based approaches to cancer gene discovery.^16^, ^17^, ^18^, ^19^ These studies showed that functional networks could be helpful in identifying cancer driver genes. However, they attempt to identify cancer driver modules consisting of a number of genes rather than individual genes crucial to cancer development. To overcome this problem, some methods prioritize the candidate genes. For instance, ContrastRank^20^ prioritizes candidate genes based on the distribution of putative deleterious mutations derived from three types of cancer data. And MUFFINN^21^ takes into account mutations in neighbor genes in a network by two different ways, showing good predictive performance in a large candidate set. ConsensusDriver^22^ is a meta‐predictor that reranks the candidate genes based on the ranking results of 18 existing methods, showing good predictive performance in a small top‐ranked candidate gene set (50). However, the false positive rates of these existing methods are still too high and thus need to be further improved.

In this study, we propose a novel method (MaxMIF) for prioritizing potential cancer driver genes based on a new maximal mutational impact function that integrates somatic mutation data and protein–protein interaction (PPI) data. Tested on six mutation datasets of Pan‐Cancer and 19 datasets of individual cancer types from TCGA, MaxMIF almost always significantly outperforms the state‐of‐the‐art tools such as MUFFINN, MutSig2.0, MutSigCV, Mutation_Assessor, and ContrastRank, in terms of the ROC (receiver operating characteristic) curve, the F1 score (harmonic average of the precision and recall), and the cumulative number of recovered known cancer genes by top‐ranked candidate genes. MaxMIF is also highly robust to various data perturbations tested. MaxMIF's ability to concentrate most likely candidate genes in a short list facilitates their experimental validations.

## 2 Results

### 2.1 Overview of MaxMIF

**Figure 1:**
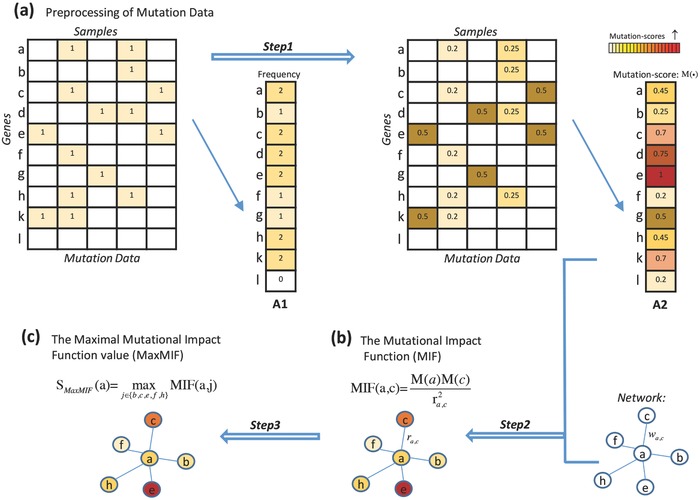
Flowchart of MaxMIF. a) The somatic mutation data matrix (rows are genes and columns are samples) is standardized by columns, then the mutation score of each gene is computed as the sum of the row. b) The mutational impact function (MIF) value of two candidate genes is computed as the product of their mutation scores divided by the square of the interaction distance between them in the PPI networks. c) For each candidate gene, the maximal MIF score is computed by considering all its neighbors in the networks. *w*_*a*,*c*_, the interaction weight between genes *a* and *c* in the PPI networks. *r*_*a*,*c*_, the “biological distance” between genes *a* and *c*, the reciprocal of *w*_*a*,*c*_.

Our MaxMIF pipeline consists of three steps (**Figure** **1**). First, we compute a mutation score for each candidate driver gene for its role in driving cancer based on somatic mutation data (Figure 1a). We designed the mutation score, such that each cancer sample in which the genes were mutated contributes equally to the score, because studies have shown that there are only a small number of driver mutation genes^5^ regardless of the total number of mutated genes in the sample. In this way, we avoid possible biases caused by samples with large number of mutated genes to stratify genes according to their resulting much different mutation scores (A1 vs A2 in Figure 1a). Second, we calculate a mutational impact function (MIF) value for each pairs of candidate genes, measuring their mutational impacts according to their relationship in PPI networks (Figure 1b). Motivated from the gravity principle^23^ (see the Supporting Information for details), two genes should have a strong mutational impact if they both have a high mutation score and are close to each other in PPI networks. Therefore, we define the MIF value of two genes to be proportional to the product of their mutation scores but inversely proportional to the square of the distance between them in PPI networks. Finally, we compute a novel maximal mutational impact function value for each candidate gene by considering all its neighbors in the PPI networks (Figure 1c) to rank the candidate genes according to their maximal mutational impact function values.

### 2.2 Performance of MaxMIF on Six Datasets of Pan‐Cancer

**Figure 2:**
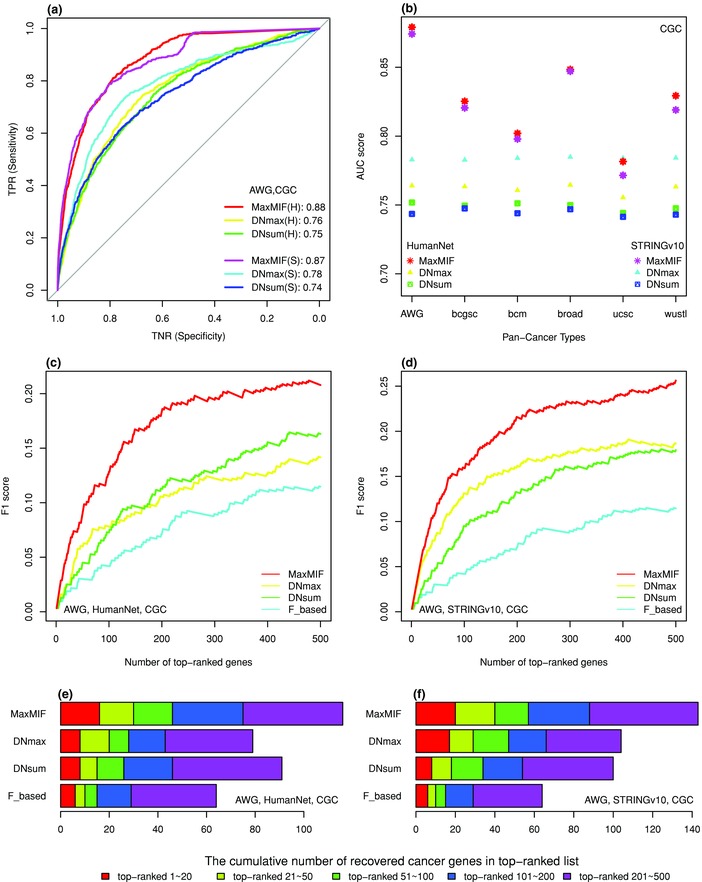
Comparison between MaxMIF and MUFFINN on the Pan‐Cancer datasets. a) ROC plots of the results of the three methods on the AWG Pan‐Cancer dataset, using the HumanNet (H) or STRINGv10 (S) networks, and the CGC reference cancer gene set. The AUC scores of the ROC curves are shown in the legends. TNR, true negative rate, represents specificity; TPR, true positive rate, represents sensitivity. b) AUC scores of the results of the three methods on the six Pan‐Cancer datasets, validated on the CGC reference cancer gene set. c,d) F1 scores as a function of the number of top‐ranked driver genes returned by the four methods on the AWG Pan‐Cancer dataset, using the HumanNet and STRINGv10 networks, respectively, and the CGC reference cancer gene set. e,f) Cumulative number of known cancer genes recovered in the indicated number of top‐ranked candidate genes on the AWG Pan‐Cancer dataset, using the HumanNet and STRINGv10 networks, respectively, and the CGC reference cancer gene set.

We first tested MaxMIF's ability to differentiate drivers from passengers in six Pan‐Cancer datasets, namely, AWG, bcgsc, bcm, broad, ucsc, and wustl, provided by different research groups from the TCGA consortium (see the details in Table S1, Supporting Information), using two independently developed PPI networks HumanNet^24^ and STRINGv10.^25^ We compared the 12 prioritizing results with those obtained by DNmax and DNsum (two algorithms in MUFFINN)^21^ using the same data and the same five reference cancer gene sets, that is, CGC (Cancer Genome Census),^26^ CGCpointMut, Rule2020,^5^ HCD,^27^ and MouseMut^28^, ^29^ (see the Supporting Information for details), with CGC being the most well‐known and confident cancer gene set. Both ROC curves (**Figure** **2**a) and AUC (area under the ROC curve) scores (Figure 2b) show that MaxMIF outperforms DNmax and DNsum in the AWG Pan‐Cancer dataset, using either the HumanNet or STRINGv10 networks validated on the CGC reference cancer gene set. Similar results were obtained in the other five Pan‐Cancer datasets validated on the CGC reference set (except ucsc, Figures S1–S5, Supporting Information) as well as when the other four reference cancer gene sets were used for validation (Figures S6–S12, Supporting Information). Furthermore, most of the P values (**Table** **1**) indicate that MaxMIF is significantly superior to DNmax and DNsum in terms of sensitivity and specificity in identifying driver genes.

**Table 1:** *P* values of the hypothesis test of ROC analyses between MaxMIF and MUFFINN on six Pan‐Cancer datasets using the HumanNet or STRINGv10 networks and the CGC reference gene set

Pan‐Cancer datasets	HumanNet		STRINGv10	
	DNmax	DNsum	DNmax	DNsum
AWG	7.61 × 10^−23^	1.02 × 10^−26^	3.01 × 10^−13^	2.37 × 10^−24^
bcgsc	3.79 × 10^−7^	1.91 × 10^−9^	3.10 × 10^−3^	8.75 × 10^−8^
bcm	9.21 × 10^−4^	7.39 × 10^−5^	1.74 × 10^−1^	1.63 × 10^−4^
broad	1.34 × 10^−12^	5.08 × 10^−16^	1.28 × 10^−6^	2.14 × 10^−14^
ucsc	3.20 × 10^−2^	4.50 × 10^−3^	7.88 × 10^−1^	2.79 × 10^−2^
wustl	1.92 × 10^−7^	2.83 × 10^−10^	6.32 × 10^−3^	5.28 × 10^−8^

We next compared MaxMIF with DNmax and DNsum for ranking their predicted driver genes. Clearly, the higher a driver gene is ranked by an algorithm, the better it performs. We examined how known cancer genes are cumulated by the 500 top‐ranked candidate genes predicted by each method, measured by the F1 score as function of the ranks, which is the harmonic average of the precision and recall. As shown in Figure 2c,d, the curves of F1 score of MaxMIF increase much faster and go up to 30% higher than those of DNmax, DNsum, and the frequency‐based approach (F_based, ranks are solely based on mutation frequency) by the end of the 500 top‐ranked genes in the AWG Pan‐Cancer dataset, indicating that the precision and recall of MaxMIF are much higher than those of the other three methods. As shown in Figure 2e,f, MaxMIF identified more known cancer genes by its 20, 50, 100, 200, and 500 top‐ranked candidate genes than did DNmax or DNsum. Similar results were obtained in the other five Pan‐Cancer datasets validated on the CGC reference set (Figures S1–S5, Supporting Information) as well as when the other four reference cancer gene sets were used for validation (Figure S13, Supporting Information). Notably, the number of known driver genes retrieved by MaxMIF in its 50 top‐ranked candidate genes was approximately the same as that by DNmax or DNsum in their 100 top‐ranked candidate genes. Besides, the number of known driver genes retrieved by MaxMIF with the STRINGv10 network in its 20 top‐ranked candidates was exactly 20, reaching prefect precision in predicting driver genes. Taken together, these results clearly show that MaxMIF consistently outperforms the other methods in prioritizing driver genes validated on the five reference gene sets in the six Pan‐Cancer datasets, thus can be used to discover unknown driver genes.

### 2.3 Performance of MaxMIF on 19 Datasets of Individual Cancer Types

**Figure 3:**
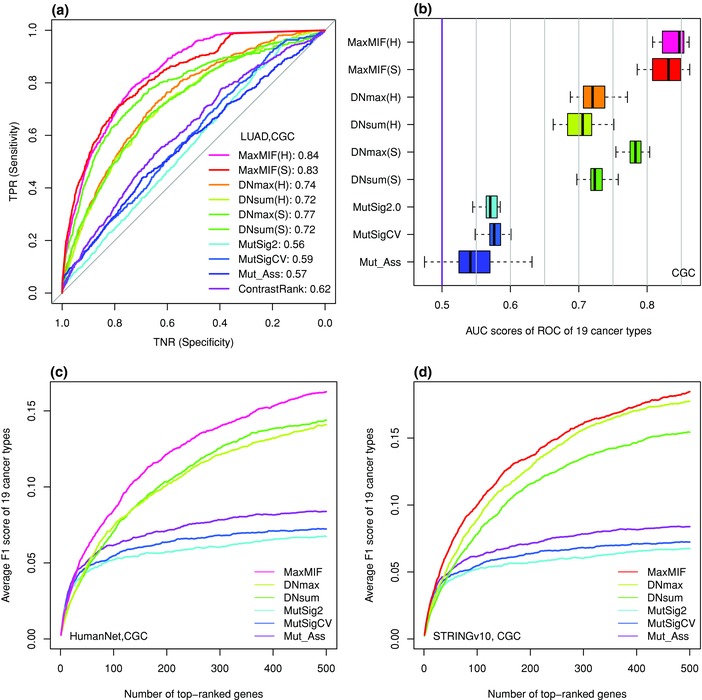
Comparison of MaxMIF with the other methods across the 19 cancer types. a) ROC curves of the results of the methods on LUAD cancer type, using the HumanNet (H) or STRINGv10 (S) networks (if network‐based) and the CGC reference cancer gene set. The AUC scores of the ROC curves are shown in the legends. b) Boxplot of the distribution of the AUC scores over the 19 cancer types. c,d) Average F1 scores as a function of the number of top‐ranked genes returned by the methods across the 19 cancer types, using the HumanNet and STRINGv10 networks, respectively (if network‐based), and the CGC reference cancer gene set.

To further evaluate MaxMIF's ability to identify responsible drive genes, we compared it with five well‐regarded methods Mutation_Assessor (Mut_Ass),^7^ MutSig2.0,^11^ MutSigCV,^12^ ContrastRank,^20^ and MUFFINN^21^ using somatic mutation datasets from 19 cancer types (see the details in Table S2, Supporting Information). Since ContrastRank is targeted at colon cancer (COAD), lung cancer (LUAD), and prostate adenocarcinomas (PRAD), we excluded it when the comparison was based on the average performance across the 19 cancer types, and further compared it with MaxMIF on the two common cancer cohorts (Figure S14, Supporting Information). As shown in **Figure** **3**a, MaxMIF, on LUAD dataset using either the HumanNet or STRINGv10 networks, outperforms all the other five methods validated on the CGC reference cancer gene set. Particularly, the MaxMIF's AUC scores computed across the 19 cancer types are much greater than those of other four methods when CGC was used as the reference gene set (Figure 3b). Similar results were obtained when validated on the other four reference gene sets, that is, CGCpointMut, Rule2020, HCD, and MouseMut (Figure S15, Supporting Information). Moreover, MaxMIF also outperforms the other methods measured by the F1 score as a function of the number of top‐ranked candidate genes on average across the 19 cancer types (Figure 3c,d and Figure S16, Supporting Information). In summary, MaxMIF significantly outperforms all the state‐of‐the‐art methods we evaluated in terms of prediction accuracy, sensitivity, and specificity.

### 2.4 Robustness of MaxMIF

**Figure 4:**
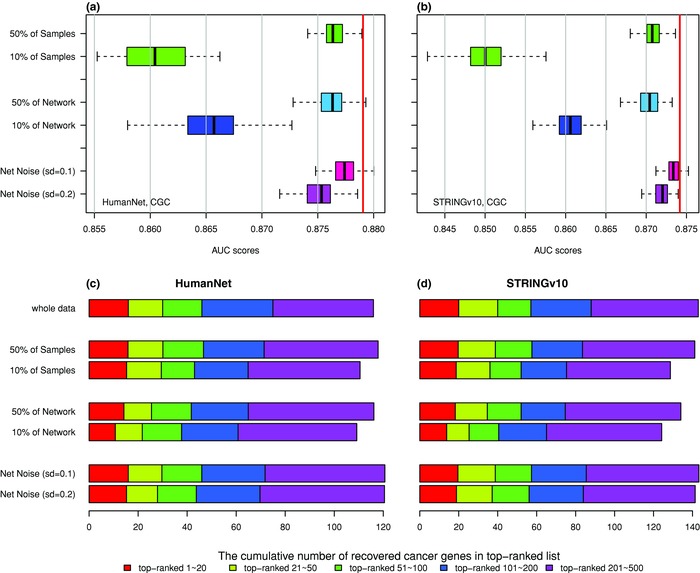
Robustness of MaxMIF. a,b) Boxplots of the effects of different data perturbation on the performance of MaxMIF measured by AUC scores over 100 repeats on the Pan‐Cancer AWG dataset using the HumanNet and STRINGv10 networks, respectively, and the CGC reference gene set. The red vertical lines represent the AUC scores by MaxMIF using all the mutation data and PPI data without noise added. c,d) Effects of different data perturbation on the performance of MaxMIF measured by the average cumulative number of known cancer genes recovered in 20, 50, 100, 200, and 500 top‐ranked candidate genes over 100 repeats on the Pan‐Cancer AWG dataset using the CGC reference gene set and the HumanNet and STRINGv10 networks, respectively. The first row represents the results of MaxMIF using all the mutation data and PPI data without noise added.

To evaluate the robustness of MaxMIF we examined it in three scenarios, each with two levels of data perturbation: (1) using only 50% and 10% of samples randomly selected from the mutation data; (2) using 50% and 10% of the pairwise interactions randomly selected from the PPI data; and (3) using all PPI data with noise added to the weights. We model the PPI noise with a Gaussian distribution of mean 0 and standard deviation 0.1 or 0.2, since the weights in the networks are standardized to a range from 0 to 1 (if a noised weight is less than 1E‐20, we assume it to be 1E‐20). Shown in **Figure** **4** are the results averaged over 100 repeats in each scenario based on the Pan‐Cancer AWG dataset and the HumanNet or STRINGv10 PPI datasets. In the first scenario, MaxMIF showed only a slight (0.03) decrease in the AUC scores, with approximately the same cumulative number of recovered cancer genes, even when only 10% of mutation data were used. In the second scenario, there was also only a slight decrease in the AUC scores and the cumulative number of recovered driver genes, even when only 10% of PPI data were used. In the last scenario, both levels of noise had almost no effect on the performance of MaxMIF. All those results demonstrate that the MaxMIF is highly robust to the size of datasets and noise in PPI data.

### 2.5 Novel Candidate Genes Predicted by MaxMIF

**Figure 5:**
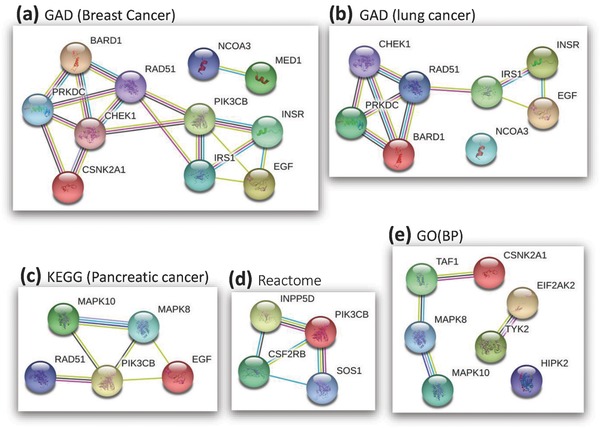
Networks of some potential novel cancer driver genes enriched in cancer related database or pathways such as GAD, KEGG, Reactome, and GO biological process. a) The network of genes enriched for “breast cancer” in GAD. b) The network of genes enriched for “lung cancer” in GAD. c) The network of genes enriched for “pancreatic cancer” in the KEGG MAPK pathway. d) The network of genes enriched for “R‐HSA‐912526” in a Reactome pathway. e) The network of genes enriched for the GO biological process “protein phosphorylation.” The networks are provided by STRING.

To evaluate MaxMIF's ability to identify potential novel cancer driver genes, we considered the genes in the 500 top‐ranked candidate lists that were predicted by MaxMIF with both HumanNet and STRINGv10 while not in CGC, resulting in 31 potential novel candidate cancer driver genes after some further screening. Intriguingly, enrichment analysis using DAVID^30^ against Genetic Association Database (GAD)^31^ that documents genes associated with complex diseases, uncovers that 28 (90.3%) of these 31 genes are included in GAD, and 18 (58.1%) genes are associated with cancer (see the details in Table S3, Supporting Information). Notably, 11 of the 31 genes are enriched for “breast cancer” (*P* value = 1.7 × 10^−7^, by Fisher's exact test, FDR = 6.2 × 10^−5^, the false discovery rate adjusted by Benjamini–Hochberg procedure for multiple hypothesis tests, **Figure** **5**a), and eight genes are enriched for “lung cancer” (*P* value = 6.7 × 10^−5^, FDR = 4.2 × 10^−3^, Figure 5b). Specifically, *PRKDC* (Figure 5a,b) ranked 17th and 23th by MaxMIF with HumanNet and STRINGv10, has been reported as an essential gene required for colorectal cancer cells.^32^ *EGF* (epidermal growth factor, Figure 5a,b), ranked 133th and 432th by MaxMIF with HumanNet and STRINGv10, plays an important role in nonsmall cell lung cancer (NSCLC).^33^ *RAD51* (Figure 5a–c), ranked 140th and 480th by MaxMIF with HumanNet and STRINGv10 is known to interact with the breast cancer driver gene *BRCA2*.^34^

Moreover, similar enrichment analysis against pathway databases KEGG,^35^ Reactome,^36^ and Gene Ontology (GO)^37^, ^38^ reveals that these 31 genes are also enriched for “pancreatic cancer” (KEGG pathway, *P* value = 9.1 × 10^−5^, FDR = 2.1 × 10^−3^, Figure 5c), “R‐HSA‐912526” (Reactome pathway, *P* value = 7.3 × 10^−5^, FDR = 1.1 × 10^−2^, Figure 5d), and “protein phosphorylation” (GO biological process, *P* value = 1.3 × 10^−4^, FDR = 9.3 × 10^−3^, Figure 5e). Specifically, *PIK3CB* (Figure 5a,c,d) ranked 286th and 291th by MaxMIF with HumanNet and STRINGv10, was hypothesized as a potential oncogene in certain cancers,^39^ and has been subsequently demonstrated as an oncogene,^40^ although it has not yet been added to the CGC list under this version. *MAPK8* (Figure 5b,e), ranked 122th and 431th by MaxMIF with HumanNet and STRINGv10, is a key kinase interacting with other kinases involved in the etiology of many cancer types.^41^

## 3 Discussion

A major challenge for distinguishing driver mutation from passenger mutation genes lies in the long‐tail distribution of the mutation frequency of genes in cancer genomes. Many methods have been developed to tackle this problem based on differential mutation frequencies, but they all suffer from low sensitivity and specificity because genes frequently mutated are not necessarily drivers. Obviously, the frequency‐based methods are biased toward genes with higher mutation frequencies and samples with more mutated genes. To overcome the limitation, we developed the MaxMIF method using the following strategies. First, to balance contributions to all the candidate genes from each sample with different numbers of mutations, we assign an equal total weight of 1 to all the mutated genes in each sample (Figure 1a). Second, to push a candidate gene with lower mutation frequency in samples to the forefront of the candidate list, we compute its mutation score as the sum of its standardized weights in the samples in which it is mutated. The gene can be otherwise ranked low by the frequency‐based method as we have shown in this study (Figures 1a and 2 and Figure S17, Supporting Information). Third, since PPI data can be very useful in distinguishing drivers from passengers,^18^, ^21^ we proposed the new metric MIF to model mutational impacts between mutated genes in PPI networks, motivated from the gravity principle.^23^ Finally, we rank a candidate gene by the maximal MIF score considering all its neighbors, which integrates the mutation data with PPI data effectively.

Comparing MaxMIF with two algorithms in MUFFINN^21^ on the six somatic mutation datasets of Pan‐Cancer, we found that MaxMIF significantly outperforms MUFFINN in all the three measures, that is, ROC, F1 score, and cumulative number of recovered cancer genes. MaxMIF is also superior to MUFFINN,^21^ MutSig2.0,^11^ MutSigCV,^12^ and Mutation_Assessor^7^ in identifying driver genes in 19 individual cancer types in terms of AUC and F1 scores. Moreover, MaxMIF also outperforms ContrastRank^20^ on three different colon, lung, and prostate cancer types. Thus, its outstanding performance is quite ubiquitous. In addition, MaxMIF is very robust to weight noise in PPI data as well as the size of mutation data and PPI data. Therefore, MaxMIF can be applied in a broad range of cases. More importantly, almost all our results indicate that MaxMIF has much higher sensitivity and specificity than the other methods in discovering cancer driver genes as measured by the ROC curves (Figures 2a and 3a). The improvement is mainly attributed to our maximal mutational impact function that subtly integrates the mutation data and PPI data. On one hand, MaxMIF can rank low passenger genes even with a higher mutation frequency such as *MLL3*, *FAT3*, and *XIRP2* (all below 14000th), which could be ranked above the top 25 by the mutation frequency‐based method. On the other hand, MaxMIF can rank high potential driver genes even with a low mutation frequency such as *EGF* and *RAD51*, *PIK3CB*, and *MAPK8* (Figure 5). Thus, MaxMIF could be used to identify unknown cancer driver genes. Indeed, by considering the non‐CGC candidates ranked by MaxMIF above the 500th with both the HumanNet and STRINGv10 datasets, we identified some potential novel driver mutation genes with strong independent evidence supports in GAD,^31^ KEGG pathway,^35^ Reactome pathway,^36^ and GO biological process.^37^, ^38^

## 4 Conclusions

We have developed a novel method MaxMIF for prioritizing potential cancer driver genes by integration of somatic mutational data and PPI data. Evaluated on multiple somatic mutation datasets, MaxMIF consistently outperforms the state‐of‐the‐art tools in predictive accuracy, sensitivity, and specificity for distinguishing drivers from passengers. MaxMIF is also highly robust to data size as well as the noise in PPI data. MaxMIF can be very useful for identifying or prioritizing cancer driver genes using an increasing number of available cancer genomic data.

## 5 Materials and Methods

*Somatic Mutation Datasets and Protein–Protein Interaction Datasets*: Six nonsilent somatic mutation (nonsense mutations, missense mutations, frame‐shift indels, splice site mutations, or stop codon read‐throughs) datasets of Pan‐Cancer (namely, AWG, bcgsc, bcm, broad, ucsc, and wustl) and 19 datasets of individual cancer types were collected from the TCGA database by UCSC Browser^42^ (https://xenabrowser.net/datapages/) (Tables S1 and S2, Supporting Information). Two independently developed PPI datasets HumanNet^24^ and STRINGv10^25^ were downloaded from their respective websites. Each of the interaction weight between two proteins was extracted and standardized with a value ranging from 0 to 1 and divided it by the largest weight. Self‐interaction loops were removed to simplify the networks. All the proteins were referred with their gene Entrez IDs from NCBI updated on May 12, 2017.

*Evaluation Criteria and Reference Cancer Gene Sets*: The performance of methods for prioritizing candidate genes was evaluated using the following criteria: the ROC analysis and AUC scores for recovering known driver genes, the F1 score and the cumulative number of known driver genes recovered in top‐ranked candidate genes. As only a limited number of top‐ranked candidate genes warrant further experimental verification, the analysis was mainly focused on the 500 top‐ranked candidates. The F1 score and the cumulative number of recovered known cancer genes were used to assess the ability of a method to concentrate real driver genes in the top‐ranked candidates. The F1 score (balanced F‐score) is the harmonic mean of precision and recall, which represents the accuracy of the binary classification. Different methods may recover different number of known cancer driver genes in their 500 top‐ranked candidates, the recall was calculated using the total number of known cancer driver genes in a reference cancer gene set to eliminate the possible inequities. The ROC analyses and statistical tests were performed using the “delong” program^43^ in the “pROC” package in R,^44^ with the null hypothesis that the two compared AUCs are the same.

To accurately assess the methods for identifying candidate driver genes, ideally, an unbiased comprehensive known cancer gene set was needed. Unfortunately, such a gold‐standard set of cancer genes is currently unavailable. Alternatively, five different cancer gene sets were collected to reduce the bias caused by using a single reference cancer gene set: (i) 616 cancer genes from the CGC,^26^ currently the most popular cancer gene set; (ii) a subset of 245 CGC cancer genes that mainly undergo somatic point mutations in various cancers (CGCpointMut); (iii) 125 cancer genes screened by the “20/20 rule” (Rule2020);^5^ (iv) 291 high‐confidence candidate genes concentrated by a rule‐based method (HCD);^27^ (v) 797 candidate cancer genes were identified as human ortholog of mouse cancer genes (MouseMut)^28^, ^29^ (see details in the Supporting Information and the overlaps of the five reference gene sets are shown in Figure S18, Supporting Information). In spite of the fact that each reference cancer gene set has a different trade‐off for accuracy, credibility, comprehensiveness, and unbiasedness, a more effective method should consistently outperform the other methods evaluated on the five reference gene sets.

*Scoring Scheme of MaxMIF: Preprocessing of Mutation Data*: The mutation data are summarized in a binary mutation matrix *M*, in which the rows represent the genes, and the columns the cancer samples (patients). For a protein‐coding gene *i*, *M*(*i*, *j*) = 1 if it has at least one nonsilent somatic mutation in sample *j*, and for a nonprotein coding gene *i*, *M*(*i*, *j*) = 1 if it has at least one mutation; and *M*(*i*, *j*) = 0 otherwise. A mutation score *M*(*i*) for each gene *i* is computed to account for the contribution of its mutations to cancer, defined as

(1) $$M\left(i\right)=\{\begin{array}{c} \sum_{k\in K_{i}}\frac{1}{N_{k}},K_{i}\neq \emptyset \\ \frac{1}{N_{\max}},K_{i}=\emptyset \end{array}$$

where *K_i_* is the set of samples in which gene *i* is mutated, *N_k_* the total number of mutated genes in sample *k*, and *N*_max _ the maximal number of mutated genes in all the samples. If gene *i* is not mutated in all the samples, that is,*K_i_* is empty, *M*(*i*) is assigned a background mutation score (BMS) that is no larger than those of any mutated genes. In this way, each sample equally contributes to the mutation score regardless of the total number of mutated genes in the samples, balancing the contributions of all the samples with different number of mutated genes (Figure S19, Supporting Information). Besides, the BMS could help to avoid missing the possible driver genes, especially when the sample size is very small (Figure S20, Supporting Information). Therefore, driver genes with a small number of mutations can still be discovered.

*The Mutational Impact Function*: To measure the impact of interactions between two mutated genes on biological functions, the MIF value was introduced between two genes *i* and *j*, motivated by the gravity principle^23^

(2) $$\mathrm{MIF}\left(i,j\right)=\frac{M\left(i\right)M\left(j\right)}{r_{ij}^{2}}$$

(3) $$r_{ij}=1/W\left(i,j\right)$$

where *M*(*i*) is the mutation score of gene *i*, and *r_ij_* the “interaction distance” between genes *i* and *j*, *W*(*i*, *j*) the interaction weight between genes *i* and *j* in the network. Thus, MIF integrates mutation information and functional relationships between the two genes, and two genes with high mutation scores and close to each other in a PPI network would have a high MIF value.

*The Maximal Mutational Impact Function*: To integrate somatic mutation data and functional interaction networks, the maximal mutational impact function value is calculated for each candidate gene *i*, *S*_MaxMIF_(*i*), defined as

(4) $$S_{\mathrm{MaxMIF}}(i)=\{\begin{array}{c} \underset{j\in J_{i}}{\max}\mathrm{MIF}\left(i,j\right),J_{i}\neq \emptyset \\ \frac{M{\left(i\right)}^{2}}{r_{\max}^{2}},J_{i}=\emptyset \end{array}$$

where *M*(*i*) is the mutation score of gene *i*, MIF(*i*, *j*) the MIF between gene *i* and *j*, *J_i_* the set of neighbors of gene *i* in the network, and *r*_max _ the largest “interaction distance” in the network. Therefore, the model uses the strongest mutational impact between the gene and its neighbors, helping to identify possible driver genes. The average MIF score over all the neighbors was considered as well, but its performance was inferior to that of the maximal MIF.

## Conflict of Interest

The authors declare no conflict of interest.

## Supporting information

Supplementary
