# Supplementary material for: MaxMIF: A New Method for Identifying Cancer Driver Genes through Effective Data Integration
Source: Adv Sci (Weinh). 2018 Jul 23;5(9):1800640. doi: 10.1002/advs.201800640 (PMC6145398; doi:10.1002/advs.201800640)
Supplement: Supplementary file 1 — Supplementary [file ADVS-5-1800640-s001.pdf]

Copyright WILEY-VCH Verlag GmbH & Co. KGaA, 69469 Weinheim, Germany, 2018.

ADVANCED SCIENCE
Open Access

Supporting Information

for *Adv. Sci.*, DOI: 10.1002/advs.201800640

MaxMIF: A New Method for Identifying Cancer Driver Genes through Effective Data Integration

Yingnan Hou, Bo Gao, Guojun Li, and Zhengchang Su**

Supplemental Material

MaxMIF: a new method for identifying cancer driver genes through effective data integration

Yingnan Hou[1,2], Bo Gao[1,2], Guojun Li[1,2,3,*], Zhengchang Su[3,*]
[1]School of Mathematics, [2]State Key Laboratory of Microbial Technology, Shandong University, Jinan 250100, P. R. China
[3]Department of Bioinformatics and Genomics, The University of North Carolina at Charlotte, 9201 University City Blvd, Charlotte, NC 28223, USA

Email addresses:

YH: houynyj@163.com

BG: gaobo19877@126.com

GL: guojunsdu@gmail.com

ZS: zsu@uncc.edu

[*]To whom correspondence should be addressed

Table of Content

Supplementary Methods

1. **TCGA data collection**
2. **Reference cancer gene sets**
3. **Evaluation criteria**
4. **Comparison between MaxMIF and the Gene Gravity Model**

Supplementary Figures
Supplementary Tables

References

Supplementary Methods

1. TCGA data collection

We collected six non-silent somatic mutation datasets of Pan-Cancer and 19 datasets of individual cancer types from the TCGA database. The Pan-Cancer datasets were combined from various TCGA cohorts, produced by different research groups named AWG, bcgsc, bcm, broad, ucsc and wustl (Supplementary Table S1). The 19 datasets of individual cancer types (Supplementary Table S2) consist of bladder urothelial carcinoma (BLCA), breast invasive carcinoma (BRCA), cervical squamous cell carcinoma & endocervical adenocarcinoma (CESC), colon & rectum adenocarcinoma (COADREAD), glioblastoma multiforme (GBM), head & neck squamous cell carcinoma (HNSC), kidney renal clear cell carcinoma (KIRC), kidney renal papillary cell carcinoma (KIRP), acute myeloid leukemia (LAML), brain lower grade glioma (LGG), lung adenocarcinoma (LUAD), lung squamous cell carcinoma (LUSC), ovarian serous cystadenocarcinoma (OV), pancreatic adenocarcinoma (PAAD), prostate adenocarcinoma (PRAD), skin cutaneous melanoma (SKCM), stomach adenocarcinoma (STAD), thyroid carcinoma (THCA), and uterine corpus endometrioid carcinoma (UCEC).

2. Reference cancer gene sets

To accurately assess the methods for identifying candidate cancer genes, ideally, we need an unbiased comprehensive known cancer gene set. Unfortunately, such a gold-standard set of cancer genes is currently unavailable. Alternatively, we collected five different cancer gene sets from different sources to minimize the possible bias caused by a single reference gene set. First, we downloaded 616 cancer genes from the Cancer Genome Census (CGC) database (v78, September 2016),[1] which is one of the world's largest and most comprehensive resources for exploring the impact of somatic mutations on cancer in human. While CGC is widely used as one of the most well-known and confident cancer gene sets, it is heavily biased towards cancer genes through chromosomal translocation (about 58.6% of CGC genes). There are many mutation types in CGC, including translocation, amplification, large deletion, frame shift, splice site, missense mutation and so on. To reduce the impact of CGC's bias caused by structural rearrangement, we generated a second reference cancer gene set containing 245 genes mutated mainly by point mutations, CGCpointMut, a subset of CGC. The third reference gene set contains 125 genes altered by intragenic mutations, promoting or driving tumorigenesis.[2] A gene was classified as an oncogene if >20% of the recorded mutations were at recurrent positions and are missense, and as a tumor suppressor gene if >20% of the recorded mutations were inactivating. The "20/20 rule" (Rule2020) is suitable as a cancer gene set, although all well-documented cancer genes far surpassed these criteria. Notably, most of those genes have been added into the updated CGC sets. The fourth reference gene set is composed of 291 high-confidence candidate driver genes (HCD) provided by a rule-based method,[3] whose most genes had signals of positive selection in at least one methods out of four: MuSiC,[4] OncodriveFM,[5] OncodriveCLUST[6] and ActiveDriver.[7] Finally, this candidate cancer gene set containing 797 orthologous genes of mouse cancer genes identified by insertional mutagenesis in mice (MouseMut)[8, 9] was for reference only as they are yet not extensively validated. The overlaps of the five reference gene sets were illustrated by a Venn diagram using the "VennDiagram" package in R (Supplementary Figure S24).[10]

3. Evaluation criteria

We evaluated the performance of the methods for prioritizing candidate genes using the following measures: the ROC analysis and AUC scores for recovering known cancer genes in the

reference sets, and the F1 score and the cumulative number of known cancer genes recovered in top-ranked candidate genes. The ROC and AUC score assess both the sensitivity and specificity of a method in distinguishing drivers from passengers at the overall level. The F1 score and the cumulative number of recovered known cancer genes in top-ranked candidates were used to assess the ability of a method to concentrate the real cancer genes in the top-ranked candidates. The F1 score combines precision and recall with equal weight, and is defined as,

$$\text{precision}=\frac{TP}{\min(N_R, N_P)},\ \text{recall}=\frac{TP}{N_R},\ \text{F1 score}=2\times\frac{precision\times recall}{precision+recall},$$

where N_P is the total number of genes that are predicted as driver cancer genes, N_R the number of genes in the reference cancer gene set, and TP the number of the reference genes in the predicted genes.

4. Comparison between MaxMIF and the Gene Gravity Model

Although our Mutational Impact Function (MIF) was motivated by the "gravitation principle" presented in the Gene Gravity Model (GGM),[11] who had clearly demonstrated that interacting proteins tend to accumulate more mutations than random protein pairs, the mutational score M(i) and the biological distance r_{ij} in the two methods were produced by totally different strategies with different biological implications. First, in MaxMIF, the mutation-score considers all somatic mutation samples while balancing their contributions, thus it represents the overall level of mutations of the gene in the samples. In contrast, in GGM the mutation-score is the cumulative mutations computed by a random walk algorithm whose restart parameter needs to be selected without standard guidelines, thus its biological meaning is intuitively unclear. Second, in MaxMIF, the biological distances between two genes is their inverse path distances in the PPI (protein-protein interaction) networks, while in GGM, it is their inverse Pearson Correlation Coefficients (PCC) of the gene expression profiles of the two genes in the PPI networks. To see whether the inverse PPI distance is better than the inverse PCC, we compared the two ways of using "biological distance" by MaxMIF with HumanNet and STRINGv10 on six cancer types as used in GGM (BRCA, GBM, HNSC, KIRC, LUAD and LUSC), together with the same gene expression profiles as used in GGM. As shown in Supplementary Figures S26 and 27, inverse PCC almost consistently underperformed inverse path distances based on either the HumanNet or the STRINGv10 PPI networks. Third, opposite to GGM, in MaxMIF the maximal MIF value of a gene instead of the average MIF value among its' neighbors in the PPI network is used as the finally score, which in our opinion better represents the functional impact on pathway level of the mutations. Most importantly, GGM collected the Cancer driver gene set (together with DNA repair gene set, Chromatin regulation factors set, Essential gene set and other cancer gene set) based on previous studies and analyzed the average Gene Gravity scores (GGS) on the level of the five gene sets, which are quite different from what we are doing.

These differences might explain the remarkable difference in the performance between the two strategies (if it makes sense that GGM is treaded as a prioritizing method). As shown in Supplementary Figures S28 and S29, MaxMIF consistently outperforms GGM across the eight cancer types (used in GGM), in terms of the ROC curves, AUC scores, F1 scores and number of

known cancer genes retrieved in their 20, 50, 100, 200, 500 top-ranked candidate genes, using either HumanNet or STRINGv10 networks validated on the CGC reference cancer gene set. Similar results were obtained when the other four reference cancer gene sets were used for validation (for AUC scores in Supplementary Figure S30 and others not shown due to space limitation).

Supplementary Figures

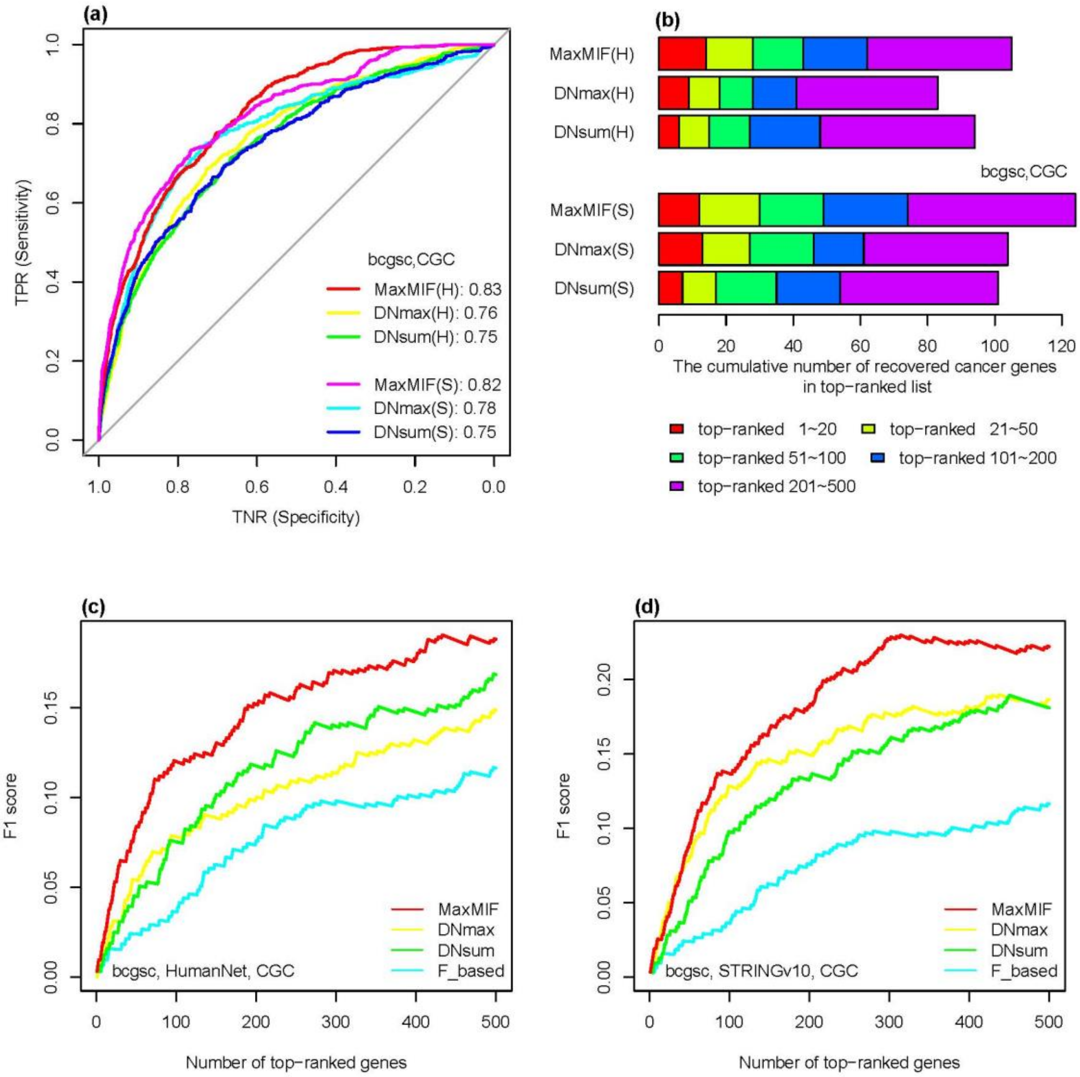

Supplementary Figure S1 Comparison between MaxMIF and MUFFINN on the bcgsc Pan-Cancer datasets. All the results of the methods were based on the bcgsc Pan-Cancer dataset, using the HumanNet (H) or STRINGv10 (S) networks, and the CGC reference cancer gene set. (**a**) ROC curves of the three methods. The AUC scores of the ROC curves are shown in the legends. TNR, true negative rate, represents specificity; TPR, true positive rate, represents sensitivity. (**b**) Cumulative number of known cancer genes recovered in the indicated number of top-ranked candidate genes. (**c, d**) F1 scores as a function of the number of top-ranked driver genes returned by the four methods.

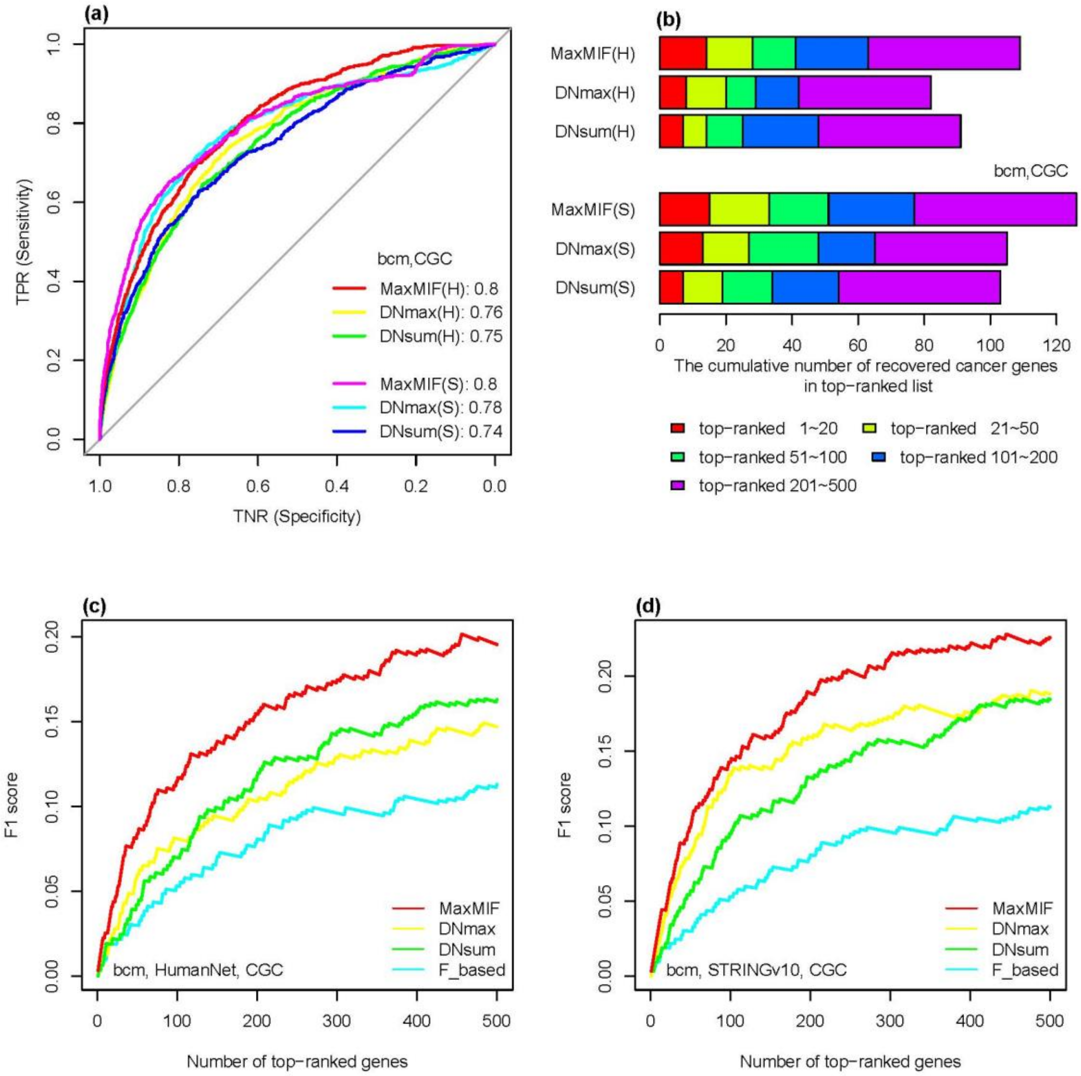

Supplementary Figure S2 Comparison between MaxMIF and MUFFINN on the bcm Pan-Cancer datasets. All the results of the methods were based on the bcm Pan-Cancer dataset, using the HumanNet (H) or STRINGv10 (S) networks, and the CGC reference cancer gene set. (**a**) ROC curves of the three methods. The AUC scores of the ROC curves are shown in the legends. TNR, true negative rate, represents specificity; TPR, true positive rate, represents sensitivity. (**b**) Cumulative number of known cancer genes recovered in the indicated number of top-ranked candidate genes. (**c, d**) F1 scores as a function of the number of top-ranked driver genes returned by the four methods.

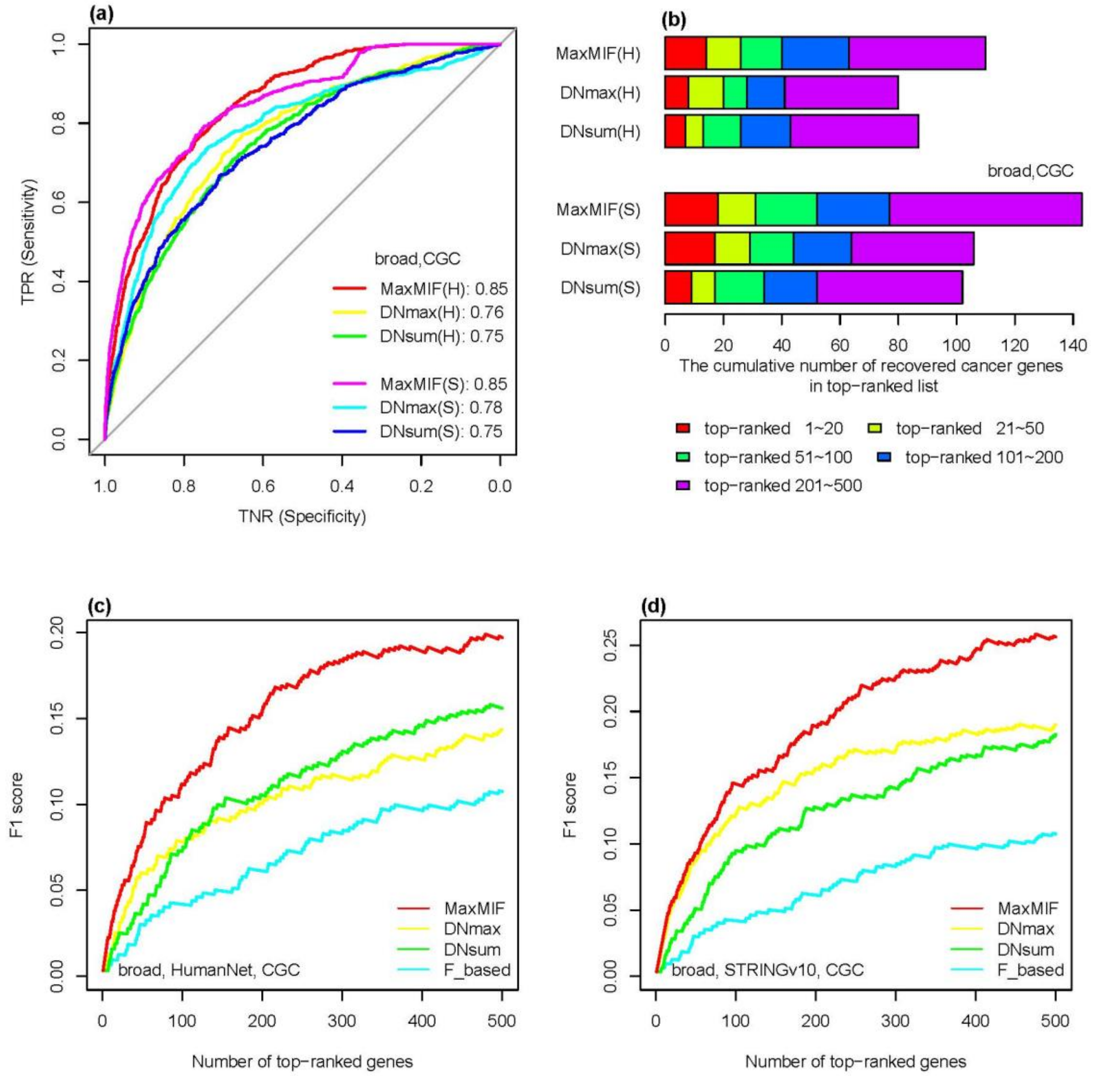

Supplementary Figure S3 Comparison between MaxMIF and MUFFINN on the broad Pan-Cancer datasets. All the results of the methods were based on the broad Pan-Cancer dataset, using the HumanNet (H) or STRINGv10 (S) networks, and the CGC reference cancer gene set. (**a**) ROC curves of the three methods. The AUC scores of the ROC curves are shown in the legends. TNR, true negative rate, represents specificity, TPR, true positive rate, represents sensitivity. (**b**) Cumulative number of known cancer genes recovered in the indicated number of top-ranked candidate genes. (**c, d**) F1 scores as a function of the number of top-ranked driver genes returned by the four methods.

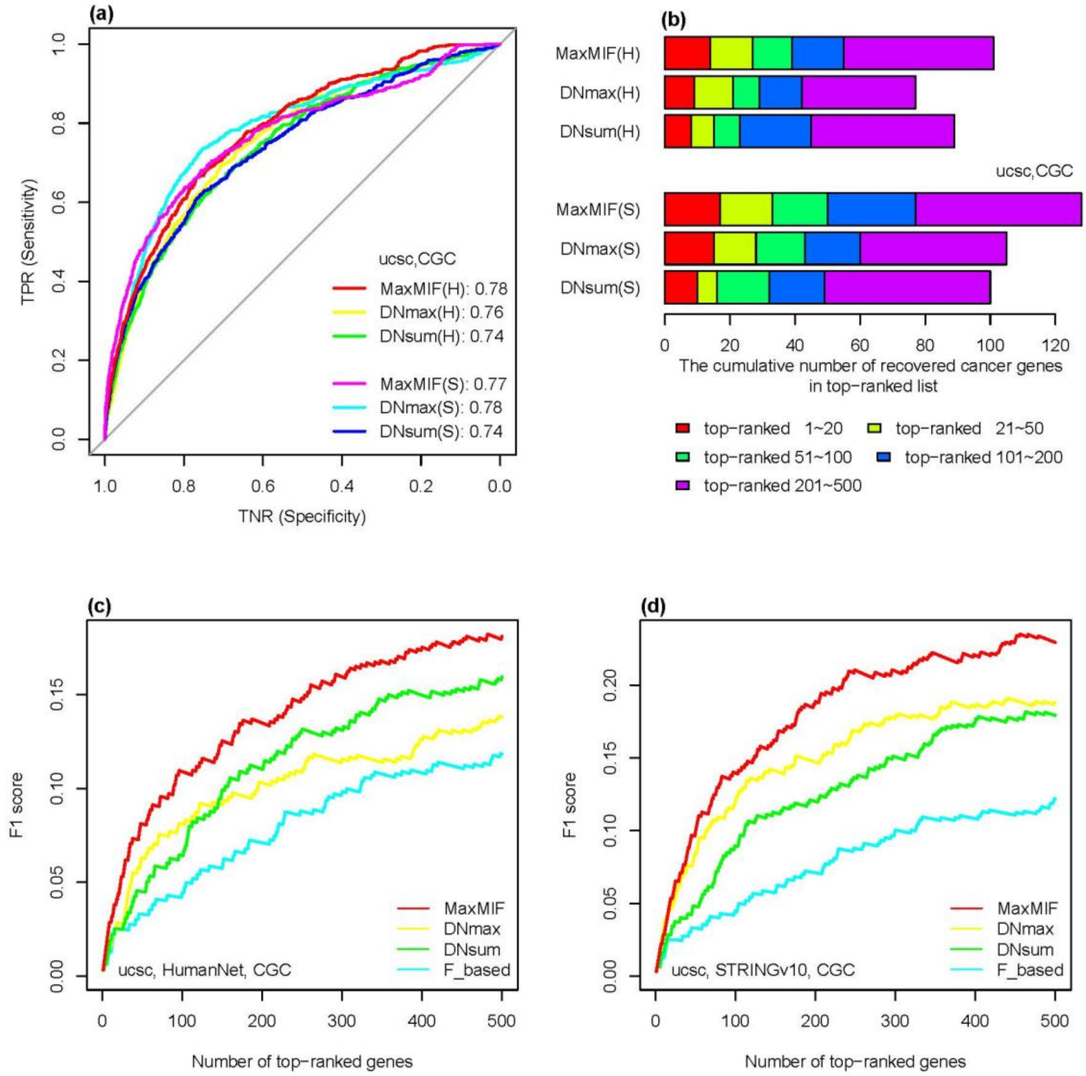

Supplementary Figure S4 Comparison between MaxMIF and MUFFINN on the ucsc Pan-Cancer datasets. All the results of the methods were based on the ucsc Pan-Cancer dataset, using the HumanNet (H) or STRINGv10 (S) networks, and the CGC reference cancer gene set. (**a**) ROC curves of the three methods. The AUC scores of the ROC curves are shown in the legends. TNR, true negative rate, represents specificity, TPR, true positive rate, represents sensitivity. (**b**) Cumulative number of known cancer genes recovered in the indicated number of top-ranked candidate genes. (**c, d**) F1 scores as a function of the number of top-ranked driver genes returned by the four methods.

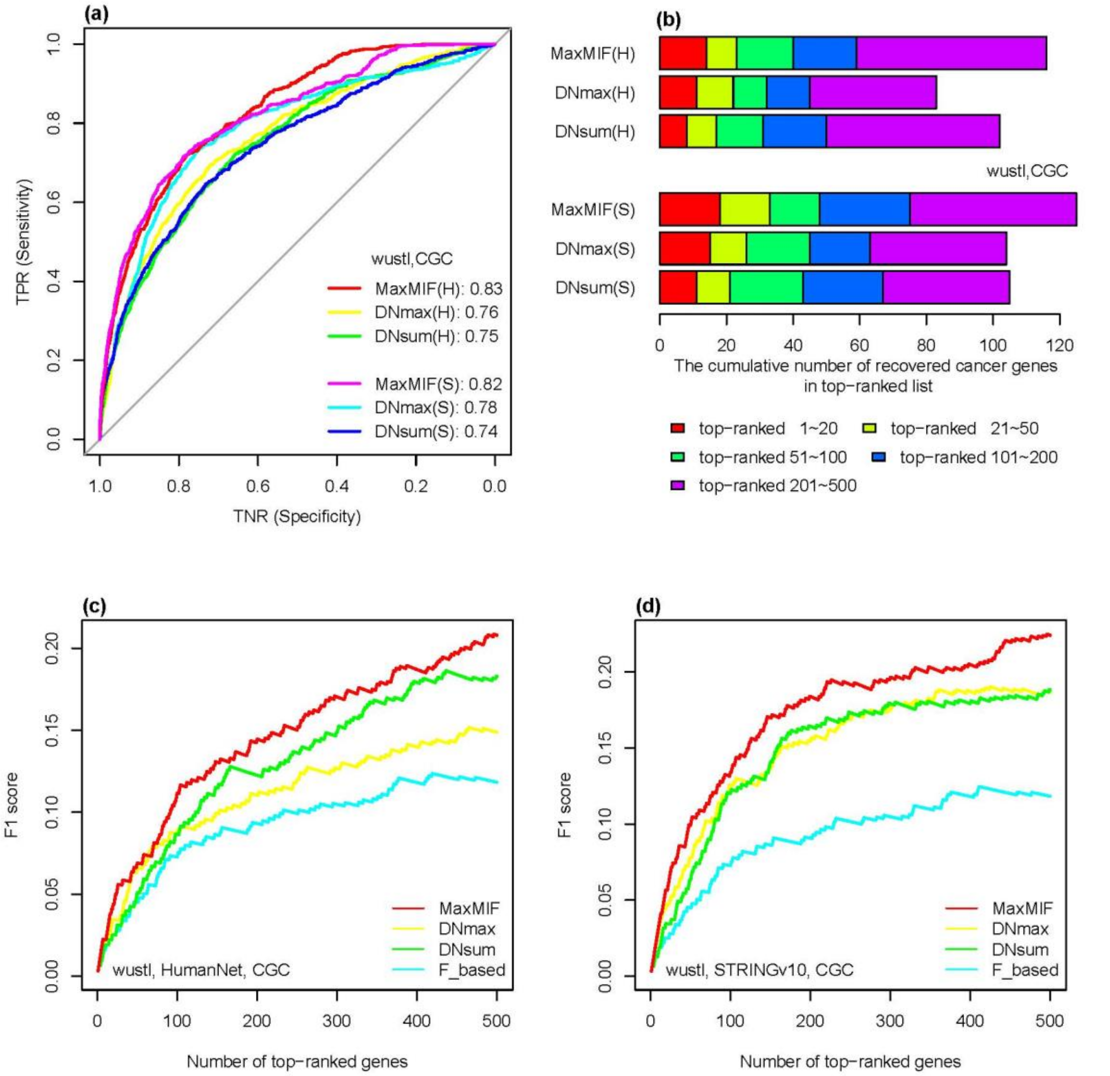

Supplementary Figure S5 Comparison between MaxMIF and MUFFINN on the wustl Pan-Cancer datasets. All the results of the methods were based on the wustl Pan-Cancer dataset, using the HumanNet (H) or STRINGv10 (S) networks, and the CGC reference cancer gene set. (**a**) ROC curves of the three methods. The AUC scores of the ROC curves are shown in the legends. TNR, true negative rate, represents specificity, TPR, true positive rate, represents sensitivity. (**b**) Cumulative number of known cancer genes recovered in the indicated number of top-ranked candidate genes. (**c, d**) F1 scores as a function of the number of top-ranked driver genes returned by the four methods.

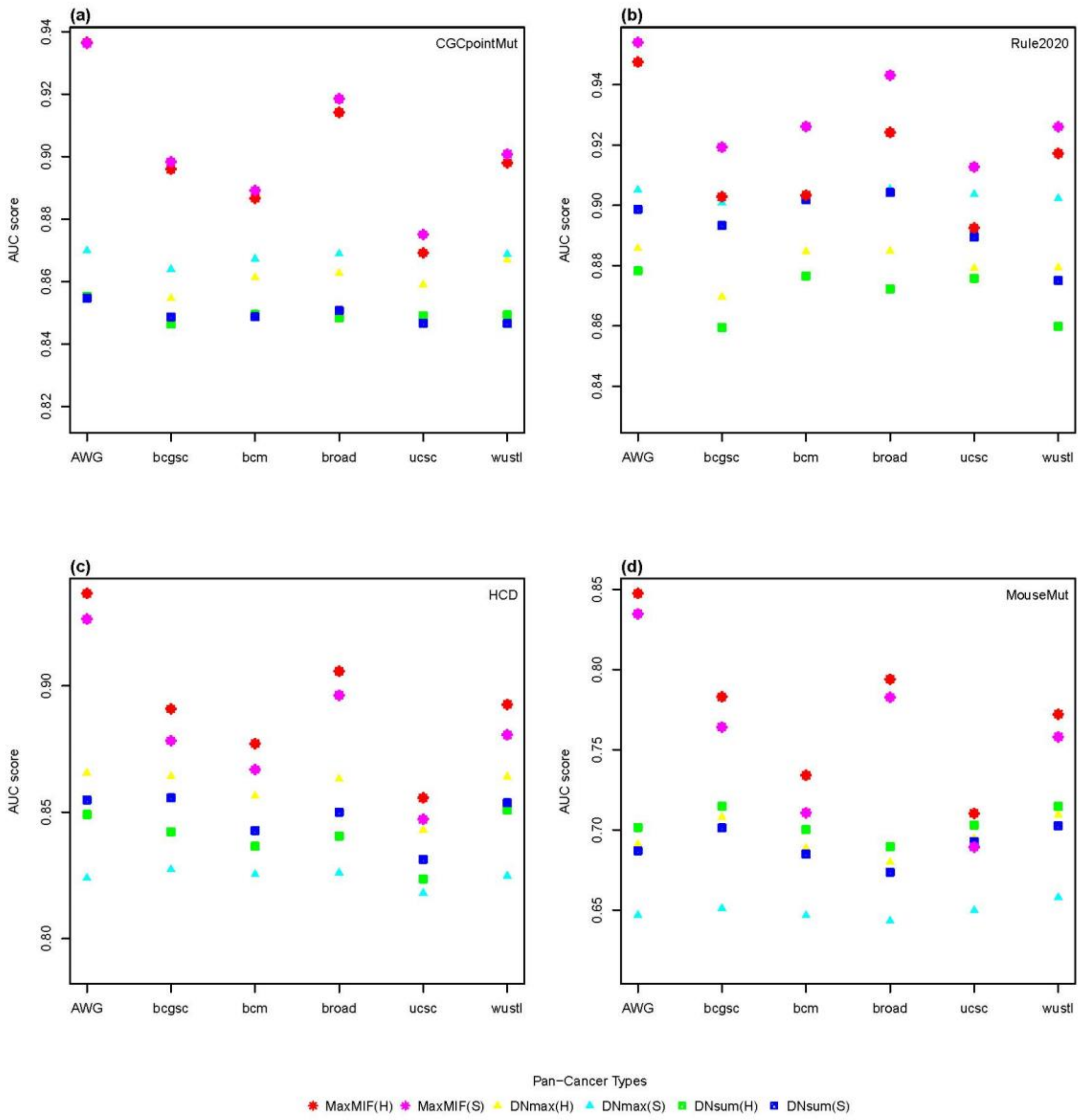

Supplementary Figure S6 Comparison of the AUC scores of the results between MaxMIF and MUFFINN (DNmax and DNsum) on the six Pan-Cancer datasets, using the HumanNet (H) or STRINGv10 (S) networks, and the four reference cancer gene sets, i.e., CGCpointMut (**a**), Rule2020 (**b**), HCD (**c**) and MouseMut (**d**).

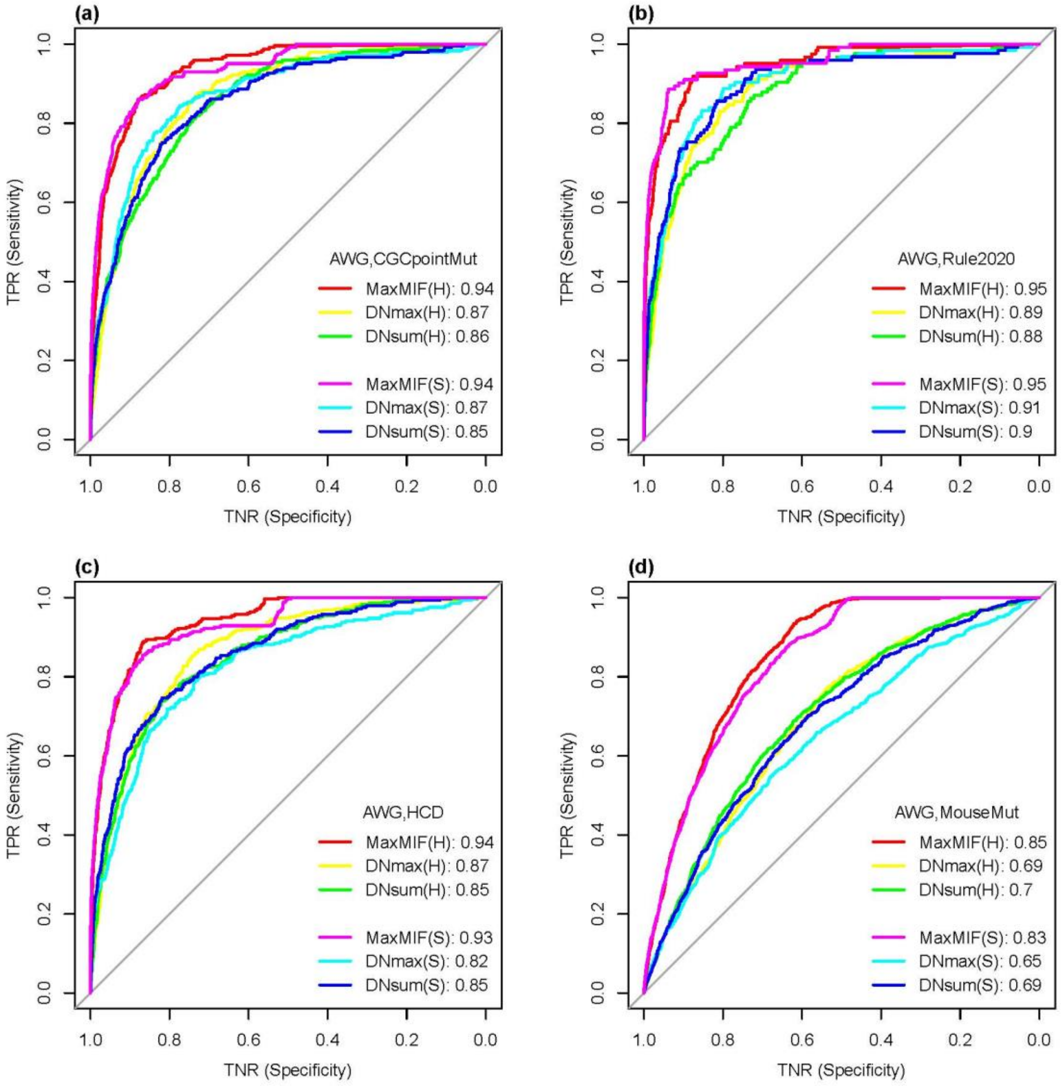

Supplementary Figure S7 Comparison of ROC curves between MaxMIF and MUFFINN (with two algorithms DNmax and DNsum) on the AWG Pan-Cancer dataset, using the HumanNet (H) or STRINGv10 (S) networks, and the four reference cancer gene sets, i.e., CGCpointMut (**a**), Rule2020 (**b**), HCD (**c**) and MouseMut (**d**). The AUC scores of the ROC curves are shown in the legends. TNR, true negative rate, represents specificity; TPR, true positive rate, represents sensitivity.

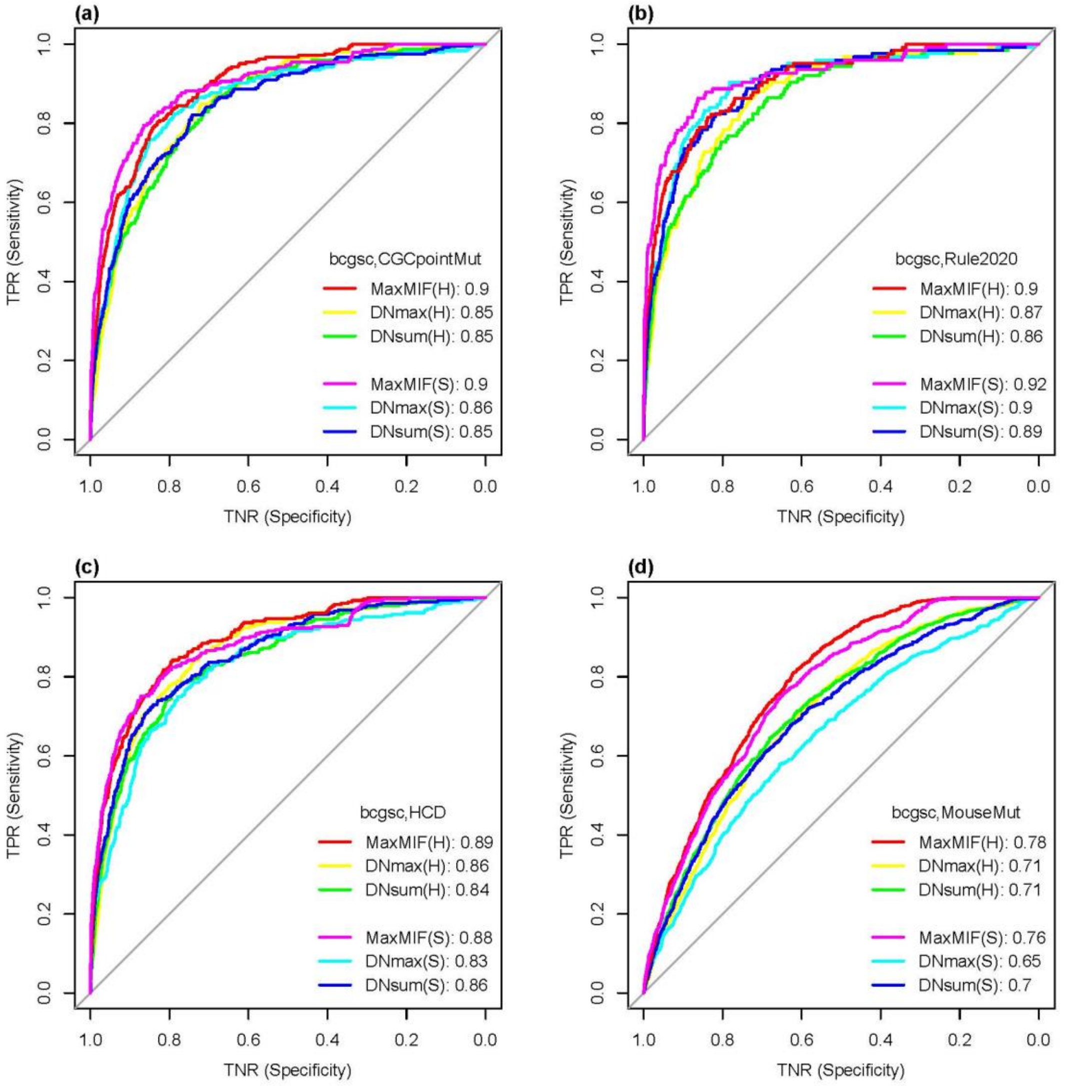

Supplementary Figure S8 Comparison of ROC curves between MaxMIF and MUFFINN (with two algorithms DNmax and DNsum) on the bcgsc Pan-Cancer dataset, using the HumanNet (H) or STRINGv10 (S) networks, and the four reference cancer gene sets, i.e., CGCpointMut (**a**), Rule2020 (**b**), HCD (**c**) and MouseMut (**d**). The AUC scores of the ROC curves are shown in the legends. TNR, true negative rate, represents specificity; TPR, true positive rate, represents sensitivity.

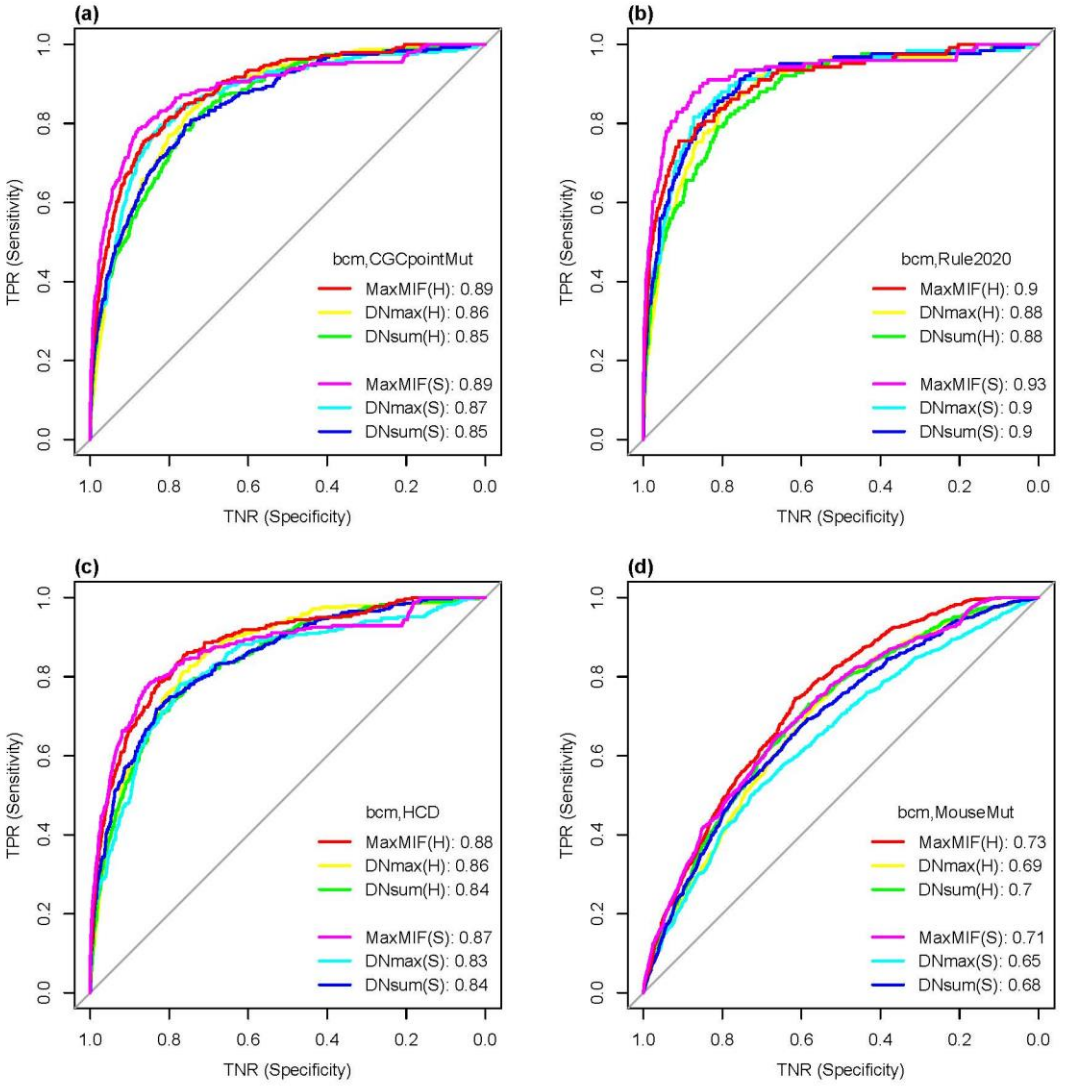

Supplementary Figure S9 Comparison of ROC curves between MaxMIF and MUFFINN (with two algorithms DNmax and DNsum) on the bcm Pan-Cancer dataset, using the HumanNet (H) or STRINGv10 (S) networks, and the four reference cancer gene sets, i.e., CGCpointMut (**a**), Rule2020 (**b**), HCD (**c**) and MouseMut (**d**). The AUC scores of the ROC curves are shown in the legends. TNR, true negative rate, represents specificity; TPR, true positive rate, represents sensitivity.

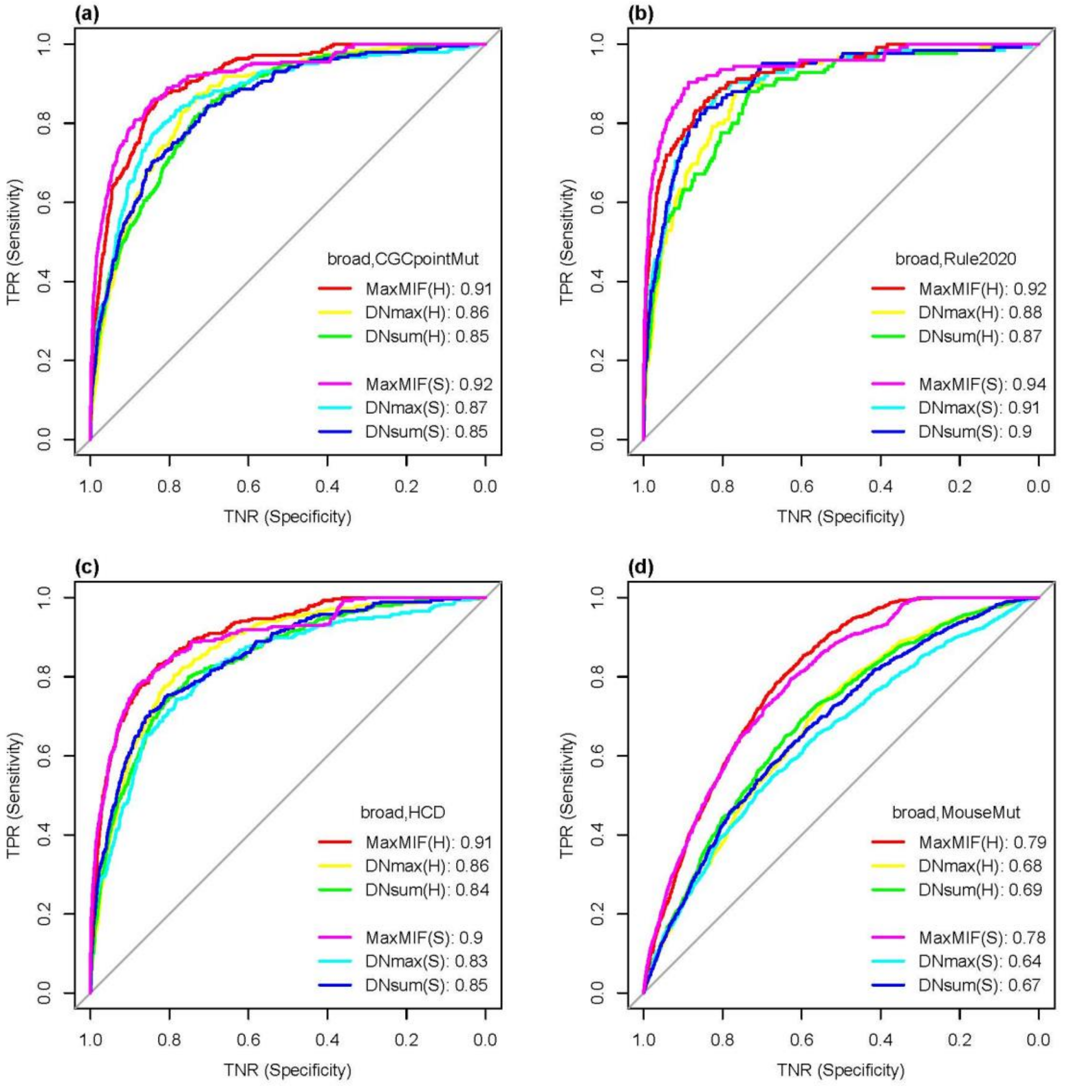

Supplementary Figure S10 Comparison of ROC curves between MaxMIF and MUFFINN (with two algorithms DNmax and DNsum) on the broad Pan-Cancer dataset, using the HumanNet (H) or STRINGv10 (S) networks, and the four reference cancer gene sets, i.e., CGCpointMut (**a**), Rule2020 (**b**), HCD (**c**) and MouseMut (**d**). The AUC scores of the ROC curves are shown in the legends. TNR, true negative rate, represents specificity; TPR, true positive rate, represents sensitivity.

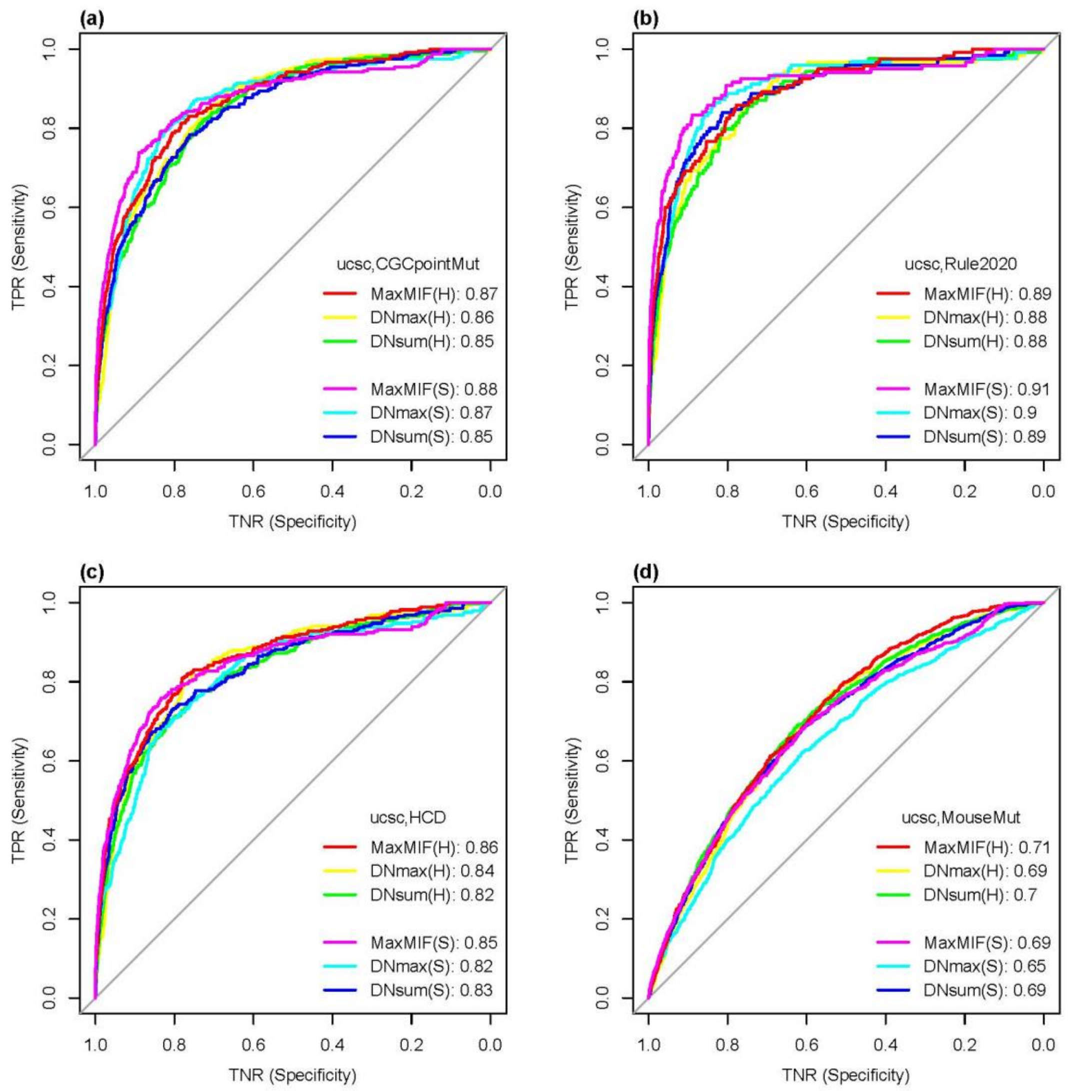

Supplementary Figure S11 Comparison of ROC curves between MaxMIF and MUFFINN (with two algorithms DNmax and DNsum) on the ucsc Pan-Cancer dataset, using the HumanNet (H) or STRINGv10 (S) networks, and the four reference cancer gene sets, i.e., CGCpointMut (**a**), Rule2020 (**b**), HCD (**c**) and MouseMut (**d**). The AUC scores of the ROC curves are shown in the legends. TNR, true negative rate, represents specificity; TPR, true positive rate, represents sensitivity.

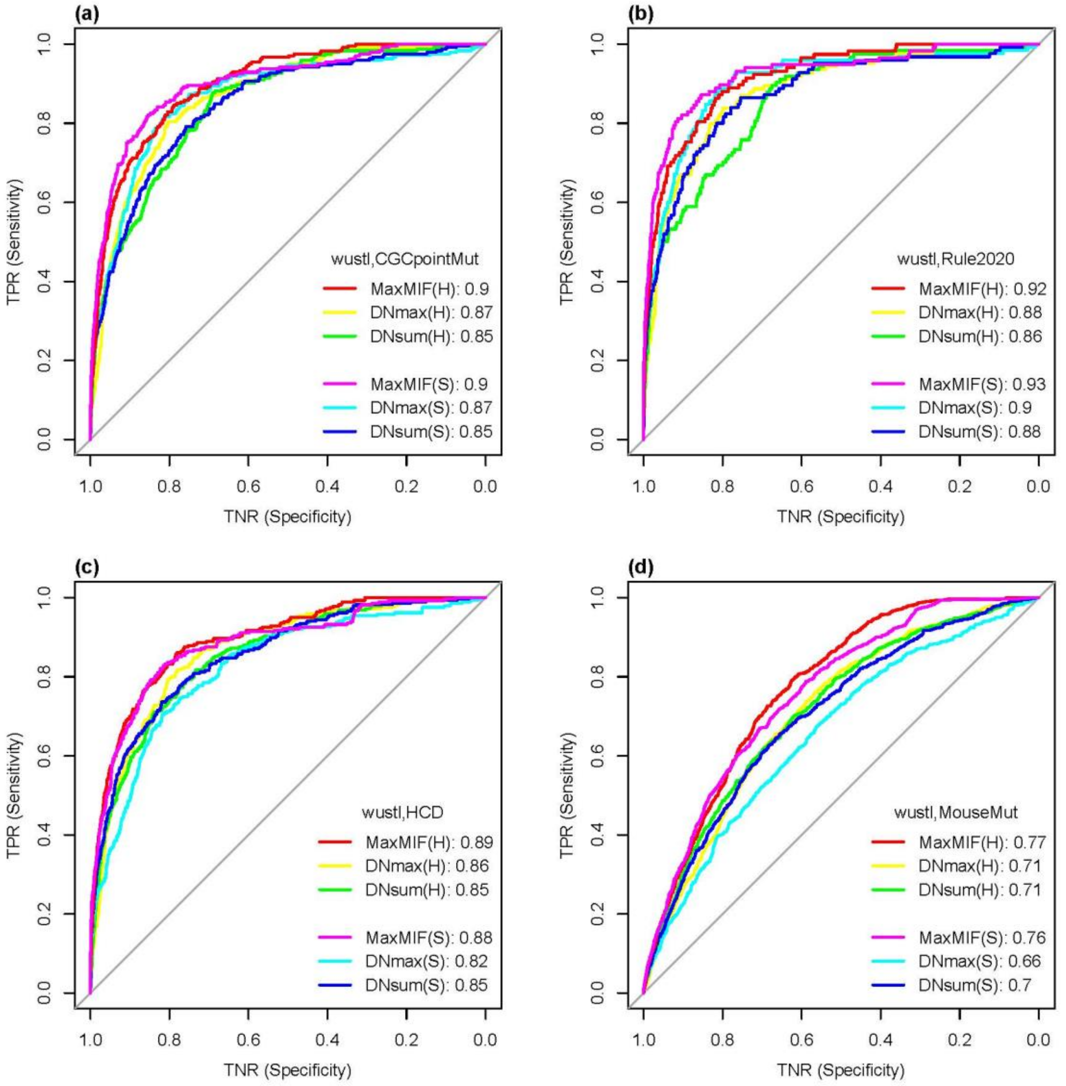

Supplementary Figure S12 Comparison of ROC curves between MaxMIF and MUFFINN (with two algorithms DNmax and DNsum) on the wustl Pan-Cancer dataset, using the HumanNet (H) or STRINGv10 (S) networks, and the four reference cancer gene sets, i.e., CGCpointMut (**a**), Rule2020 (**b**), HCD (**c**) and MouseMut (**d**). The AUC scores of the ROC curves are shown in the legends. TNR, true negative rate, represents specificity, TPR; true positive rate, represents sensitivity.

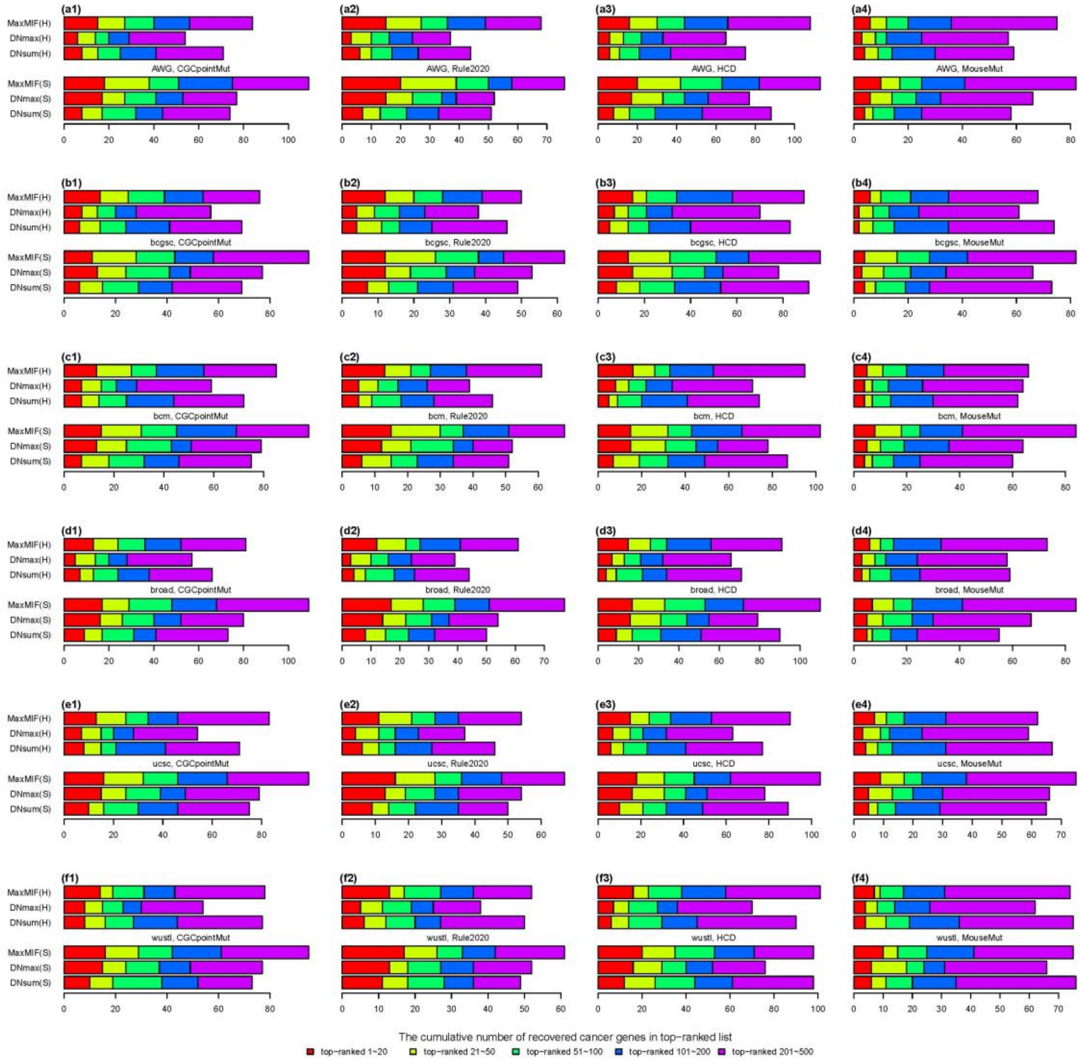

Supplementary Figure S13 Comparison of the cumulative number of known cancer genes recovered in the indicated number of top-ranked candidate genes between MaxMIF and MUFFINN (with two algorithms DNmax and DNsum). The results are based on the six Pan-Cancer dataset, i.e., AWG (**a**), bcgsc (**b**), bcm (**c**), broad (**d**), ucsc (**e**) and wustl (**f**), using the HumanNet (H) or STRINGv10 (S) networks, and the four reference cancer gene sets, i.e., CGCpointMut (**a-f1**), Rule2020 (**a-f2**), HCD (**a-f3**) and MouseMut (**a-f4**).

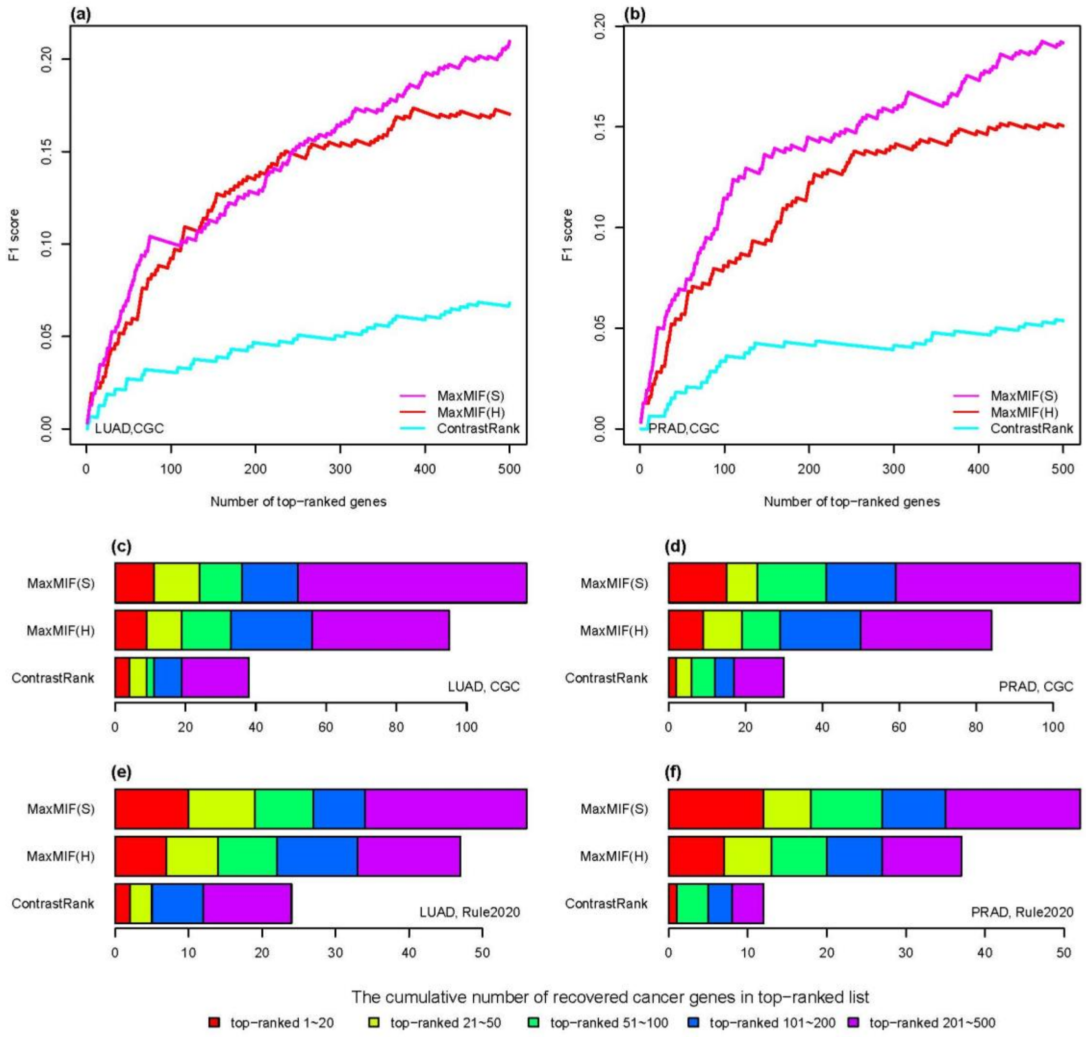

Supplementary Figure S14 Comparison between MaxMIF and ContrastRank. (**a, b**) F1 scores as a function of the number of top-ranked driver genes returned on the cancer cohorts LUAD and PRAD, using the HumanNet (H) or STRINGv10 (S) networks, and the CGC reference cancer gene set. Cumulative number of known cancer genes recovered in the indicated number of top-ranked candidate genes on the cancer cohorts LUAD and PRAD, using the HumanNet (H) or STRINGv10 (S) networks, and the CGC (**c, d**) and Rule2020 (**e, f**) reference cancer gene sets.

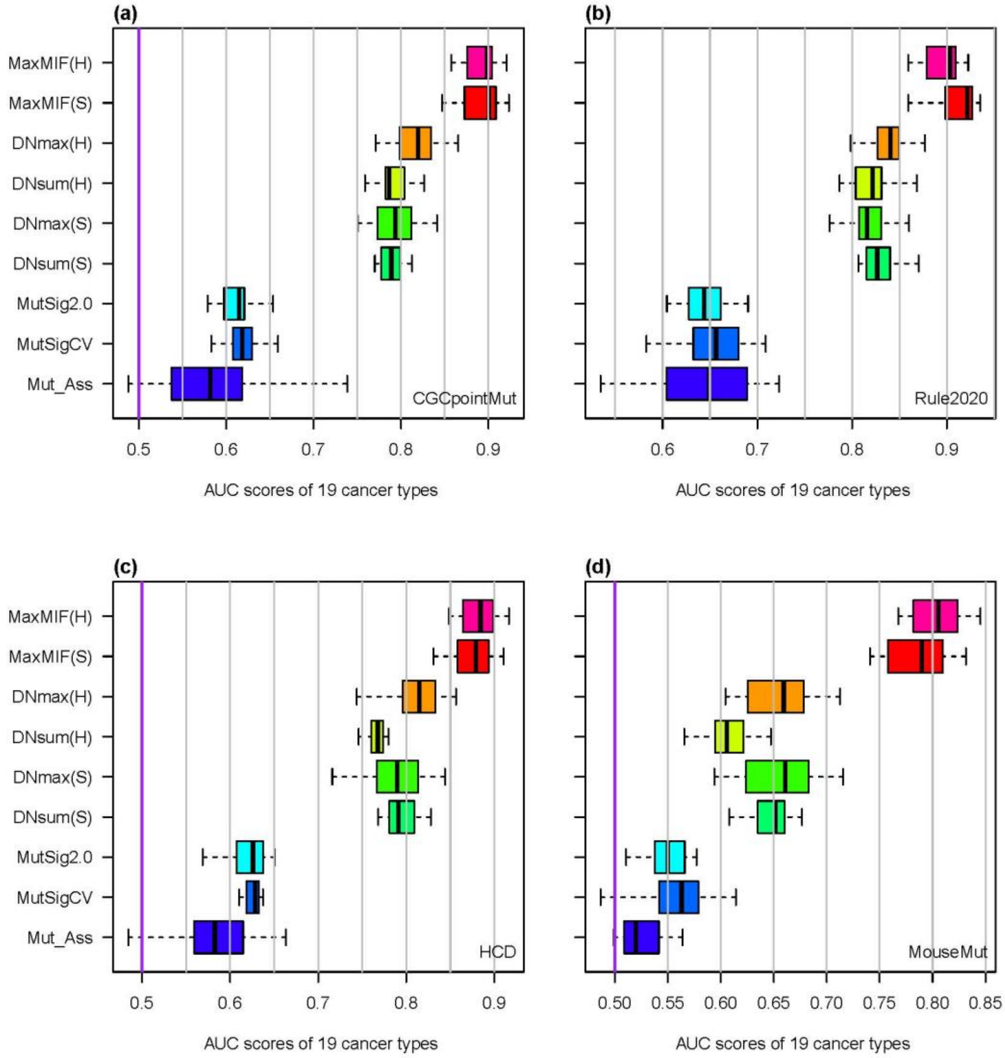

Supplementary Figure S15 Comparison of the AUC scores over the 19 datasets among MaxMIF with the other methods, i.e., MUFFINN (with two algorithms DNmax and DNsum), MutSig2.0, MutSigCV, and Mutation_Assessor (Mut_Ass), using the HumanNet (H) or STRINGv10 (S) networks if method is network-based, and the four reference cancer gene sets, i.e., CGCpointMut (**a**), Rule2020 (**b**), HCD (**c**) and MouseMut (**d**).

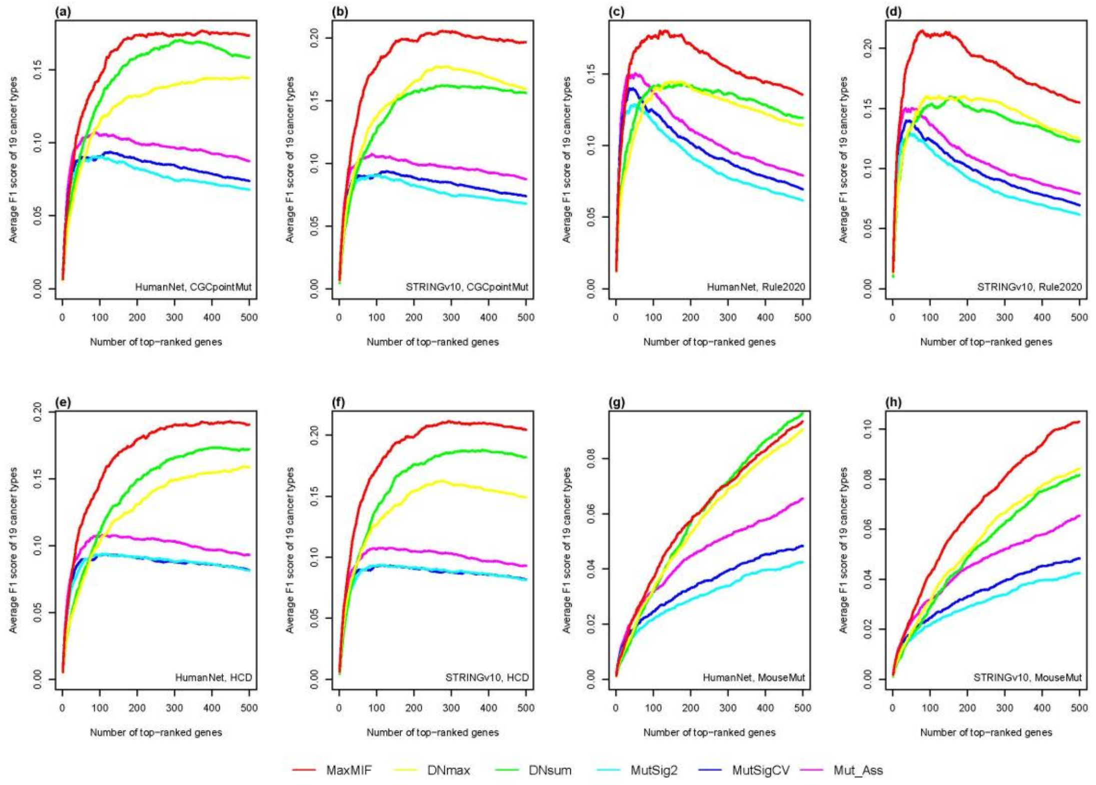

Supplementary Figure S16 Comparison of the average F1 scores as a function of the number of top-ranked genes returned among MaxMIF with the other methods, i.e., MUFFINN (with two algorithms DNmax and DNsum), MutSig2.0, MutSigCV, and Mutation_Assessor (Mut_Ass), across the 19 datasets, using the HumanNet (H) or STRINGv10 (S) networks if method is network-based, and the four reference cancer gene sets, i.e., CGCpointMut (**a**, **b**), Rule2020 (**c**, **d**), HCD (**e**, **f**) and MouseMut (**g**, **h**).

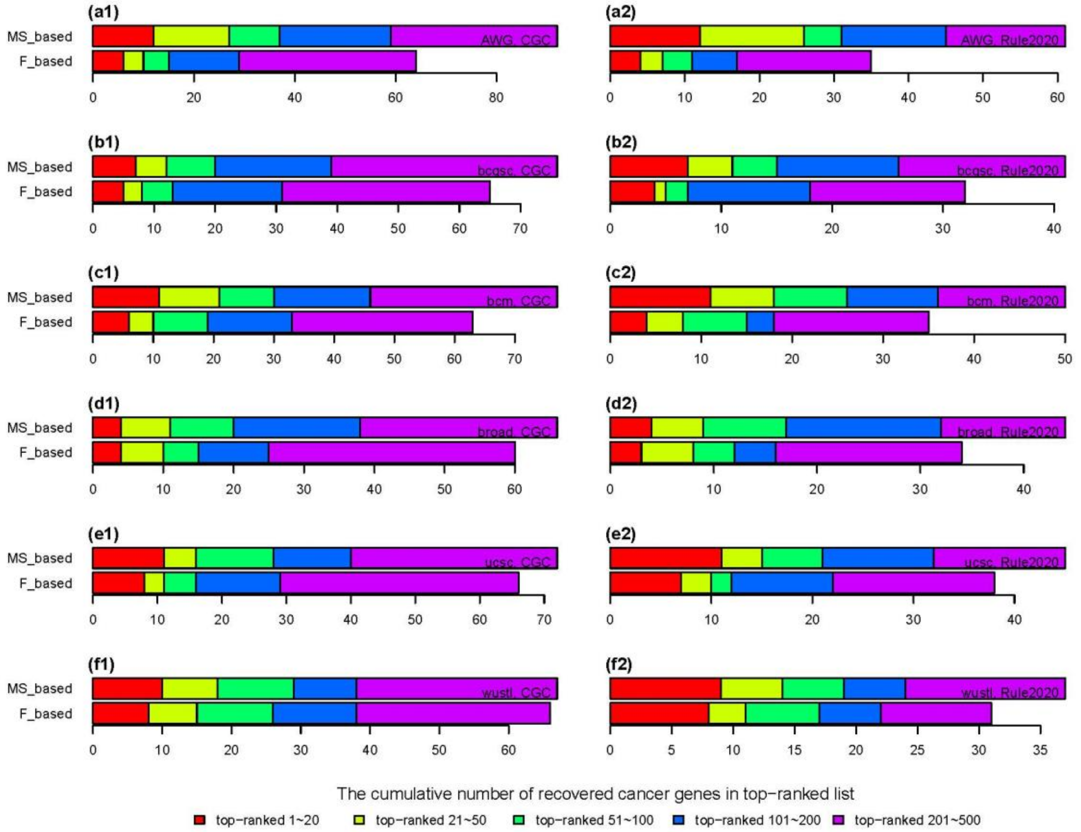

Supplementary Figure S17 Comparison of the cumulative number of known cancer genes recovered in top-ranked genes between using mutation-score (MS_based) and mutation frequency (F_based), based on the six Pan-Cancer datasets namely AWG, bcgsc, bcm, broad, ucsc and wustl, using the CGC and Rule2020 reference gene sets.

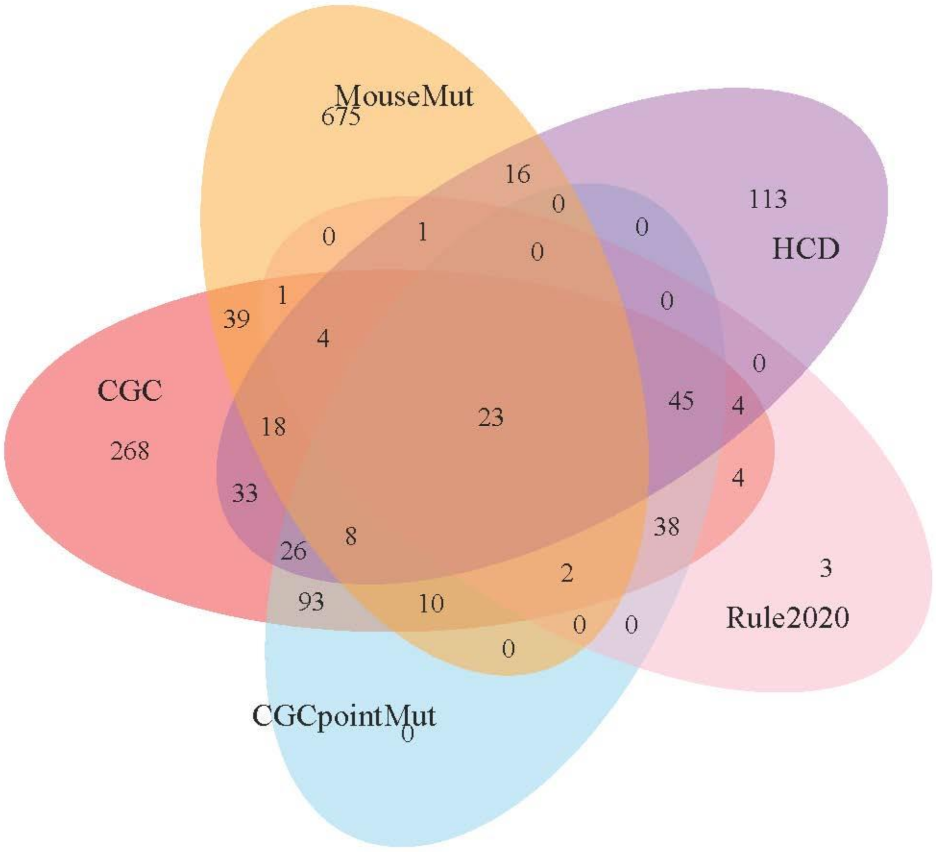

Supplementary Figure S18 The overlaps of the five reference gene sets i.e., CGC, CGCpointMut, Rule2020, HCD and MouseMut.

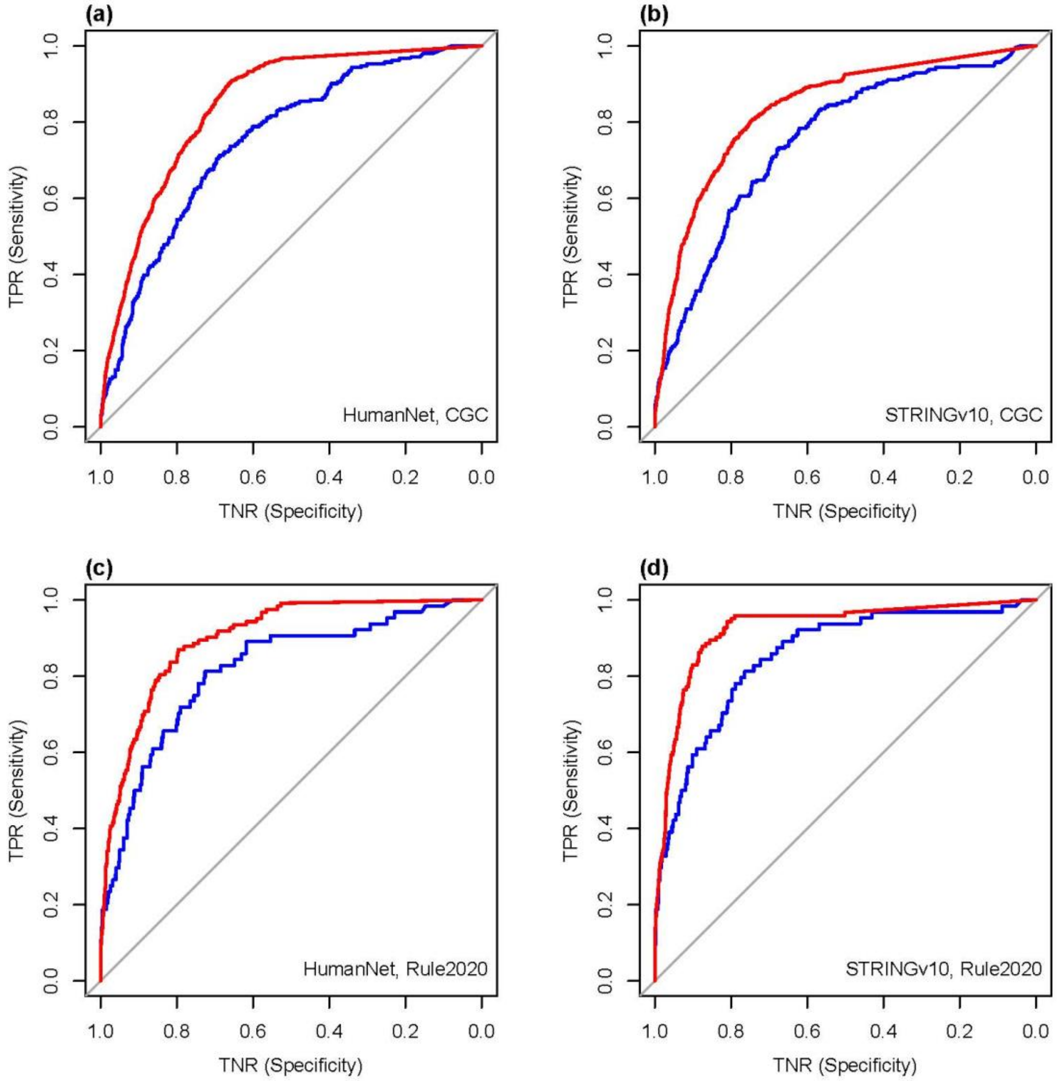

Supplementary Figure S19 Comparison of the ROC curves of MaxMIF with (red) or without (blue) a background mutation-score (BMS) based on a mutation dataset of 30 sample using the HumanNet (**a, c**) or STRINGv10 (**b, d**) networks, and the CGC (**a**, **b**) and Rule2020 (**c, d**) reference gene sets.

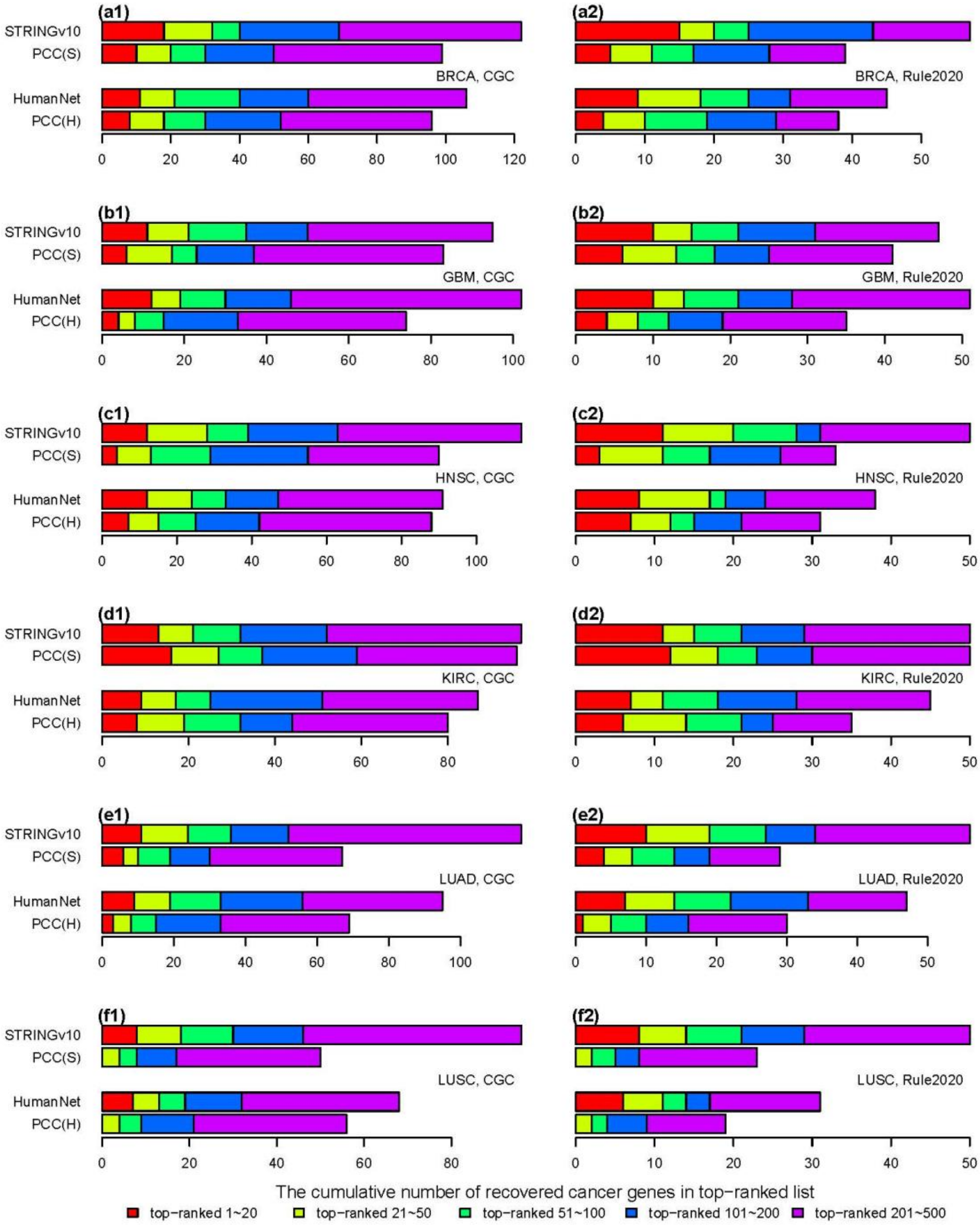

Supplementary Figure S20 Comparison between different biological distances used in the MaxMIF model. The inverse PPI distance, and the inverse Pearson correlation coefficients of the corresponding gene expression profiles of the two genes in the STRINGv10 (PCC(S)) or HumanNet (PCC(H)) were compared in MaxMIF on six cancer types used in GGM (BRCA, GBM, HNSC, KIRC, LUAD and LUSC), in term of the cumulative number of known cancer genes recovered in top-ranked genes using the CGC and Rule2020 reference gene sets.

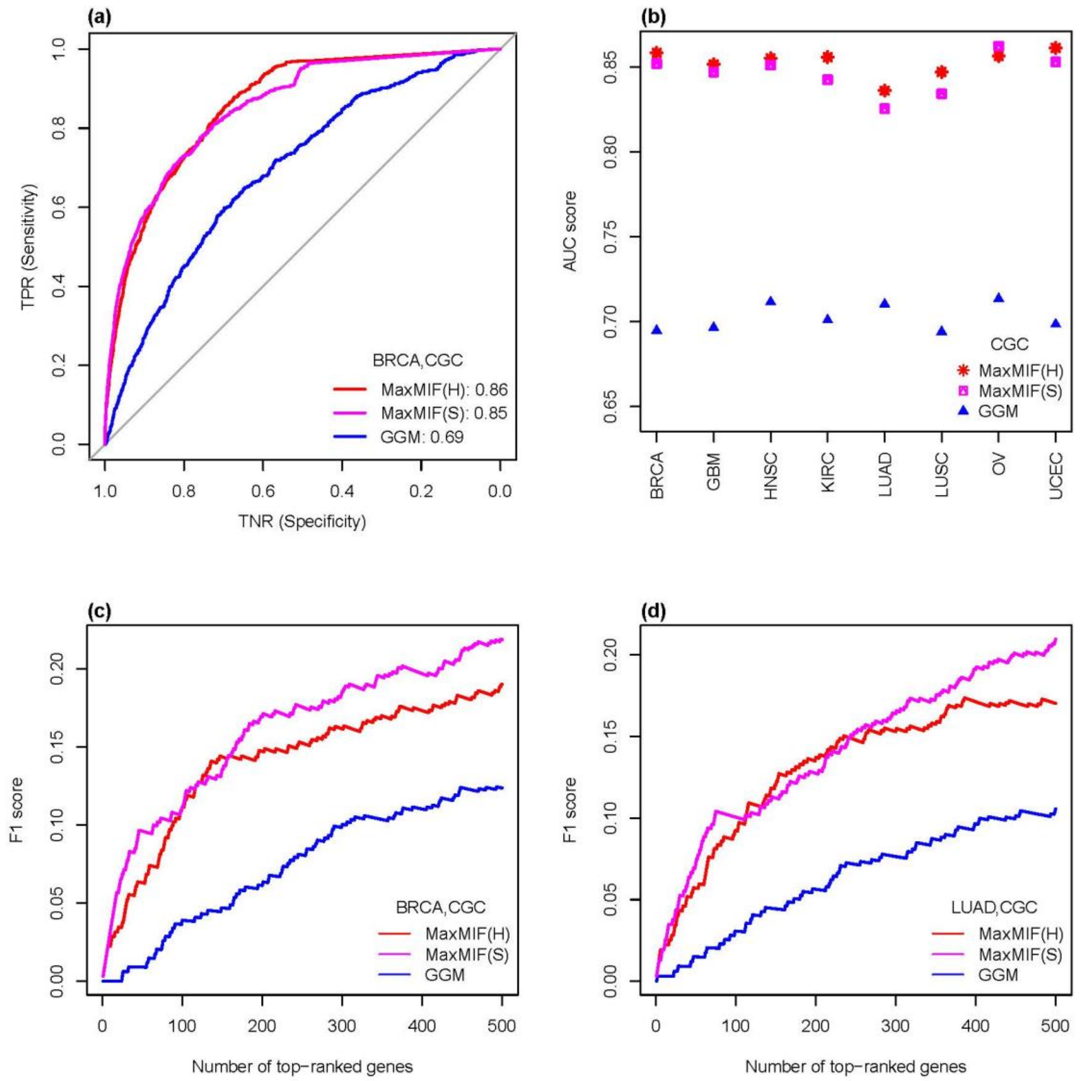

Supplementary Figure S21 Comparison of ROC, AUC score and F1 score between MaxMIF and GGM. (**a**) ROC plots of the results of the two methods on BRCA, using the HumanNet (H) or STRINGv10 (S) networks, and the CGC reference cancer gene set. The AUC scores of the ROC curves are shown in the legends. TNR, true negative rate, represents specificity; TPR, true positive rate, represents sensitivity. (**b**) AUC scores of the results of the two methods on the eight cancer types (BRCA, GBM, HNSC, KIRC, LUAD, LUSC, OV and UCEC), validated on the CGC reference cancer gene set. (**c, d**) F1 scores as a function of the number of top-ranked driver genes returned by the two methods on BRCA and LUAD, using the HumanNet (H) or STRINGv10 (S) networks, and the CGC reference cancer gene set.

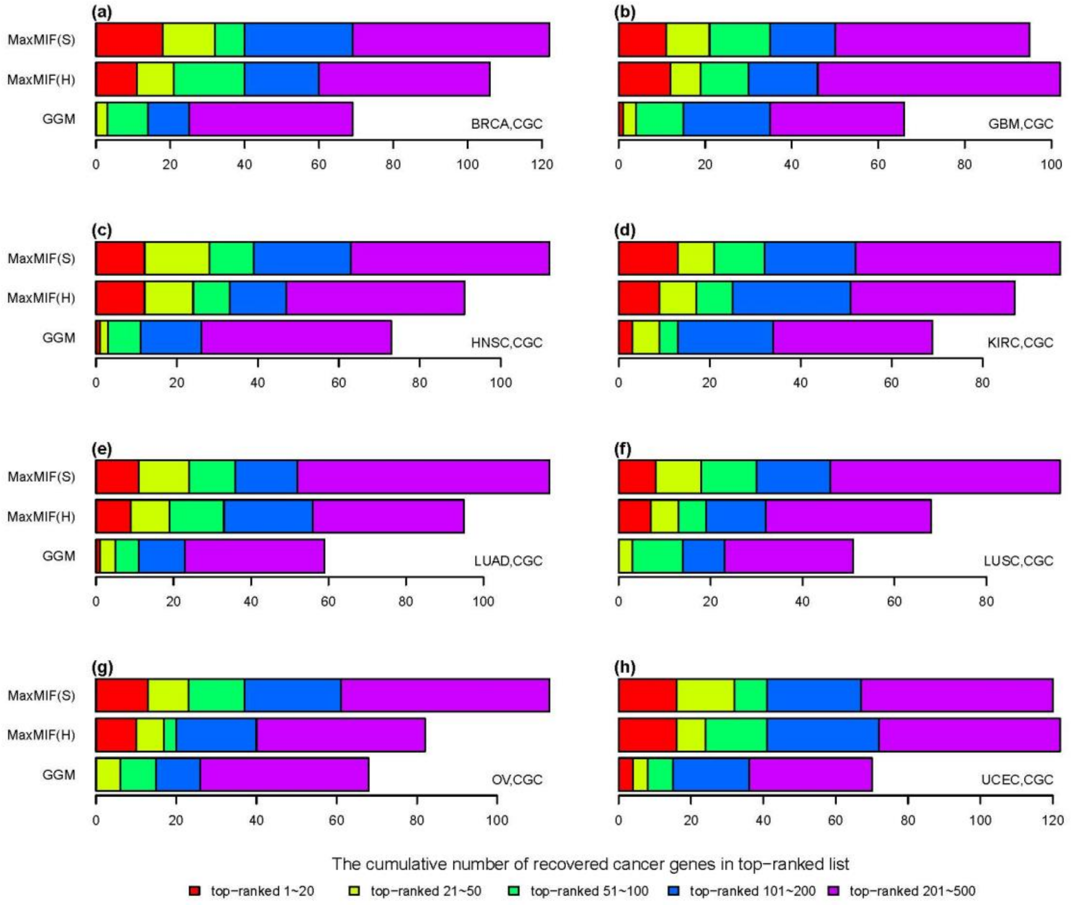

Supplementary Figure S22 Comparison of the cumulative number of known cancer genes recovered in top-ranked genes between MaxMIF and GGM, based on the eight cancer types used in GGM (BRCA, GBM, HNSC, KIRC, LUAD, LUSC, OV and UCEC), using the HumanNet (H) or STRINGv10 (S) networks if method is network-based, and the CGC reference gene set.

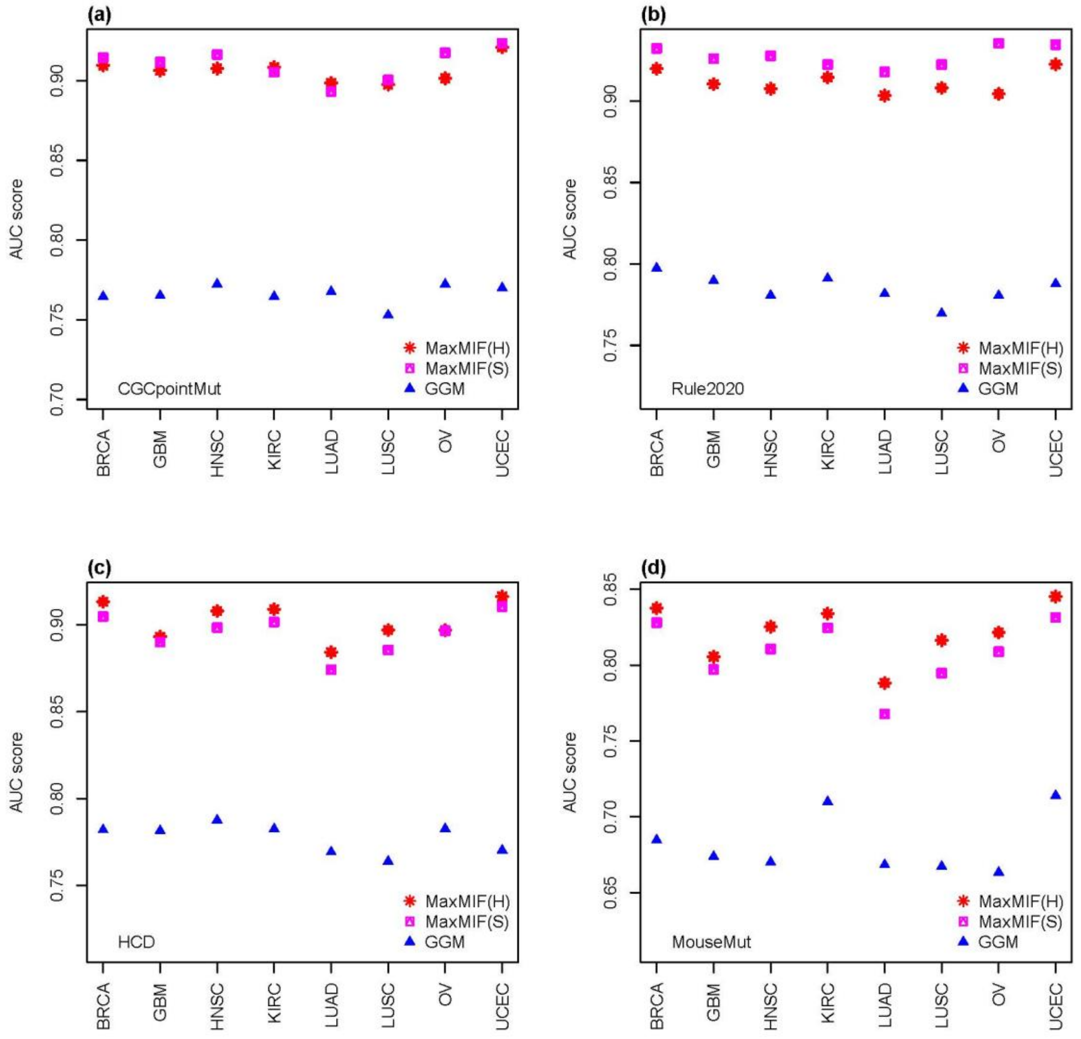

Supplementary Figure S23 Comparison of the AUC scores between MaxMIF and GGM on the eight cancer types. Cumulative number of known cancer genes recovered in the indicated number of top-ranked candidate genes, using the HumanNet (H) and STRINGv10 (S) networks, respectively, and the four reference cancer gene sets, i.e., CGCpointMut (**a**), Rule2020 (**b**), HCD (**c**) and MouseMut (**d**). The eight cancer types are BRCA, GBM, HNSC, KIRC, LUAD, LUSC, OV and UCEC.

Supplementary Tables

Supplementary Table S1 Summary of the six non-silent somatic mutation datasets of Pan-Cancer from TCGA.

Datasets	Sizes	Organizations	Methods	Version
AWG	4429	TCGA PANCANCER Analysis Working Group	_ _	2017-09-08
bcgsc	3219	Michael Smith Genome Sciences Centre (British Columbia Genome Sciences Centre	the BCGSC pipeline method	2016-08-17
bcm	4144	Baylor College of Medicine Human Genome	the Baylor pipeline	2017-10-16

		Sequencing Center	method	
broad	6333	Broad Institute Genome Sequencing Center	the MuTect method	2016-08-17
ucsc	2685	University of California Santa Cruz GDAC	the RADIA method	2016-08-17
wustl	1024	Washington University Genome Center	the WashU pipeline method	2017-10-16

Supplementary Table S2 Summary of the 19 somatic non-silent mutation datasets of individual cancer types from TCGA.

**Cohorts**	**Team**	**Sizes**	**Cancer types**	**Version**
BLCA	broad	396	bladder urothelial carcinoma	2018-01-12
BRCA	AWG	771	breast invasive carcinoma	2017-09-08
CESC	wustl	194	cervical squamous cell carcinoma & endocervical adenocarcinoma	2018-01-10
COADREAD	AWG	224	colon & rectum adenocarcinoma	2017-09-08
GBM	AWG	291	glioblastoma multiforme	2017-09-08
HNSC	AWG	306	head & neck squamous cell carcinoma	2017-09-08
KIRC	AWG	417	kidney renal clear cell carcinoma	2017-09-08
KIRP	bcm	282	kidney renal papillary cell carcinoma	2018-01-12
LAML	AWG	196	acute myeloid leukemia	2017-09-08
LGG	bcm	289	brain lower grade glioma	2018-01-12
LUAD	broad	543	lung adenocarcinoma	2018-01-12
LUSC	AWG	178	lung squamous cell carcinoma	2017-09-08
OV	AWG	316	ovarian serous cystadenocarcinoma	2017-09-08
PAAD	bcgsc	147	pancreatic adenocarcinoma	2018-01-12
PRAD	broad	499	prostate adenocarcinoma	2018-01-12
SKCM	bcm	344	skin cutaneous melanoma	2018-01-12
STAD	bcm	379	stomach adenocarcinoma	2018-01-12
THCA	broad	504	thyroid carcinoma	2018-01-12
UCEC	AWG	248	uterine corpus endometrioid carcinoma	2017-09-08

Supplementary Table S3 Potential novel cancer genes predicted by MaxMIF are enriched in GAD (Genetic Association Database). P values were computed using the Fisher's exact test, and FDR (false discovery rate) were the adjusted P values by Benjamini-Hochberg correction for multiple hypothesis tests.

**Terms**	**Count**	**P value**	**FDR**	**Genes (Entrez ID)**
Breast Cancer	11	1.67E-07	6.22E-05	1457, 5291, 580, 1111, 3667, 5888, 3643, 8202, 1950, 5591, 5469
breast cancer	9	6.95E-06	1.30E-03	5599, 580, 1111, 3667, 5888, 8202, 1950, 5591, 5469
epithelial ovarian cancer	6	2.33E-05	2.17E-03	580, 1111, 5888, 3643, 8202, 1950
lung cancer	8	6.71E-05	4.17E-03	580, 1111, 3667, 5888, 3643, 8202, 1950, 5591
Bladder Cancer	8	7.86E-05	4.18E-03	580, 1111, 3667, 5888, 3643, 8202, 1950, 5591
Colorectal Cancer	7	1.99E-04	8.23E-03	1457, 2908, 1111, 3667, 5888, 3643, 1950
prostate cancer	7	5.24E-04	1.76E-02	2908, 3667, 5888, 8202, 1950, 5591, 5469

Lung Cancer	7	1.16E-03	3.53E-02	580, 1111, 3667, 5888, 3643, 8202, 1950
brain cancer	3	3.91E-03	8.24E-02	5888, 1950, 5591
esophageal adenocarcinoma	5	5.65E-03	9.58E-02	580, 1111, 3667, 5888, 1950
ovarian cancer	5	6.80E-03	1.05E-01	5888, 7297, 8202, 1950, 5610
Brain Neoplasms|Glioma	3	8.25E-03	1.16E-01	5888, 1950, 5591
Adenocarcinoma|pancreatic neoplasm|Pancreatic Neoplasms	2	3.28E-02	3.06E-01	3667, 8471
ovarian cancer	3	3.63E-02	3.26E-01	580, 5888, 1950
Carcinoma, Papillary|Thyroid Neoplasms	2	4.28E-02	3.49E-01	5888, 5591

References

[1] S. A. Forbes, D. Beare, H. Boutselakis, S. Bamford, N. Bindal, J. Tate, C. G. Cole, S. Ward, E. Dawson, L. Ponting, *Nucleic acids research* **2016**, *45*, D777.

[2] B. Vogelstein, N. Papadopoulos, V. E. Velculescu, S. Zhou, L. A. Diaz, K. W. Kinzler, *science* **2013**, *339*, 1546.

[3] D. Tamborero, A. Gonzalez-Perez, C. Perez-Llamas, J. Deu-Pons, C. Kandoth, J. Reimand, M. S. Lawrence, G. Getz, G. D. Bader, L. Ding, *Scientific reports* **2013**, *3*, 2650.

[4] N. D. Dees, Q. Zhang, C. Kandoth, M. C. Wendl, W. Schierding, D. C. Koboldt, T. B. Mooney, M. B. Callaway, D. Dooling, E. R. Mardis, *Genome research* **2012**, *22*, 1589.

[5] A. Gonzalez-Perez, N. Lopez-Bigas, *Nucleic acids research* **2012**, *40*, e169.

[6] D. Tamborero, A. Gonzalez-Perez, N. Lopez-Bigas, *Bioinformatics* **2013**, *29*, 2238.

[7] J. Reimand, O. Wagih, G. D. Bader, *Scientific reports* **2013**, *3*.

[8] K. M. Mann, J. M. Ward, C. C. K. Yew, A. Kovochich, D. W. Dawson, M. A. Black, B. T. Brett, T. E. Sheetz, A. J. Dupuy, D. K. Chang, *Proceedings of the National Academy of Sciences* **2012**, *109*, 5934.

[9] H. N. March, A. G. Rust, N. A. Wright, J. ten Hoeve, J. de Ridder, M. Eldridge, L. van der Weyden, A. Berns, J. Gadiot, A. Uren, *Nature genetics* **2011**, *43*, 1202.

[10] H. Chen, P. C. Boutros, *BMC bioinformatics* **2011**, *12*, 35.

[11] F. Cheng, C. Liu, C.-C. Lin, J. Zhao, P. Jia, W.-H. Li, Z. Zhao, *PLoS computational biology* **2015**, *11*, e1004497.
